# Fully Printable Manufacturing of Miniaturized, Highly Integrated, Flexible Evaporation‐Driven Electricity Generator Arrays

**DOI:** 10.1002/advs.202413779

**Published:** 2024-12-17

**Authors:** Qun Liu, Panwang Guo, Xinyu Zhang, Hehe Ren, Jing Liang, Quancai Li, Weinan Tang, Wei Wu

**Affiliations:** ^1^ Laboratory of Printable Functional Materials and Printed Electronics School of Physics and Technology Wuhan University Wuhan 430072 P. R. China

**Keywords:** flexible electronics, flexible evaporation‐driven electricity generator, portable and wearable electronics, scalable integrated fabrication, screen printing

## Abstract

Harvesting sustainable clean energy from natural water evaporation holds great promise to provide continuous power for portable and wearable electronics. However, poor portability and complex fabrication processes hinder the low‐cost and large‐scale integration of flexible evaporation‐driven electricity generators (FEEGs). Herein, a fully‐printed flexible evaporation‐driven generator (PFEEG) is developed. Utilizing custom‐formulated functional inks, the asymmetric structures, current collectors, and hygroscopic water storage units can be manufactured by a patternable, scalable, and layer‐by‐layer deposition technique of screen printing. Thus, a PFEEG unit (0.5 cm × 1 cm × 38 µm) can generate a voltage of ≈0.8 V over a wide relative humidity (RH) range from 20% to 90%, and a maximum power density of 1.55 µW cm^−2^ at 70% RH. An array of 200 PFEEGs connected in series or parallel can produce voltages up to 152.41 V or a current up to 1.02 mA. Furthermore, the scalable PFEEG array can not only be seamlessly connected with the printed flexible circuit but can also be integrated with a humidity sensor and display arrays to form a self‐powered printed flexible sensing system. This work presents a practical strategy for continuous power supply of portable and wearable electronics.

## Graphical abstract

Harvesting sustainable clean energy from natural water evaporation holds great promise to provide continuous power for portable and wearable electronics. A novel manufacturing process for fully‐printed flexible evaporation‐driven electricity generators with high output power density, small size, and outstanding integrability is proposed to be a practical strategy for green energy supply of portable and wearable electronics for the first time.

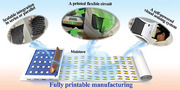

## 1 Introduction

To promote the development of next‐generation portable and wearable electronics, it is necessary to develop green and flexible energy harvesting devices to achieve their continuous energy supply.^[^ ^1^, ^2^, ^3^ ^]^ Flexible evaporative‐driven electricity generators (FEEGs) offer a promising approach to meet the long‐term power supply needs of flexible electronic devices. Because they can continuously obtain clean energy from natural water evaporation under ambient conditions.^[^ ^4^, ^5^, ^6^ ^]^ However, FEEGs are currently unable to provide long‐term power supply for portable and wearable electronics due to challenges such as portability, long‐term work, and large‐scale integration.

Typically, FEEGs are fabricated by depositing functionalized nanomaterials onto flexible substrates. The functionalized nanomaterials are stacked together to form micro/nanochannels. When a water source comes into contact with the functionalized nanomaterials, an electrical double layer (EDL) is formed at the water‐solid interface. The evaporation of water drives the spontaneous formation of capillary water flow in the micro/nanochannels, thereby directing the transport of charges. This process continuously converts environmental thermal energy into clean electricity.^[^ ^7^, ^8^, ^9^ ^]^ On the one hand, FEEGs require a enough water source either by being fixed in a water reservoir or through continuously adding water droplets manually to maintain continuous electricity generation.^[^ ^10^, ^11^, ^12^ ^]^ These water sources not only severely limit the portability and practicality of FEEGs, but also hinder their large‐scale production. On the other hand, FEEGs are typically composed of single functionalized nanomaterials, resulting in lower output power density.^[^ ^13^, ^14^ ^]^ Currently, many studies have demonstrated that the output power density can be improved through microstructural modulation. For example, the graphene oxide (GO)‐reduced graphene oxide (rGO) asymmetric structure is constructed by using the drop‐coating method to deposit GO and rGO at both ends of the carbon nanotube/polyurethane composite foam, respectively, and the current output of the FEEG can be boosted by optimizing the reduction degree of the rGO.^[^ ^15^ ^]^ Alternatively, utilizing a chemical gradient reduction strategy, GO deposited on a cotton fabric substrate via a dip‐coating method is gradient‐reduced along a unidirectional gradient to fabricate an asymmetric structure with the continuously gradient‐distributed rGO. By optimizing the time for chemical gradient reduction, the internal resistance of the FEEG is regulated, and the current output is enhanced simultaneously.^[^ ^16^ ^]^ Although these methods involving chemical processing of functionalized nanomaterials to build asymmetric structures can increase the power density of FEEG, they still cannot meet the power supply needs of portable and wearable electronic devices. It requires serial or parallel integration of multiple FEEGs to achieve the desired electric output performance. However, the aforementioned approaches for constructing asymmetric structures cannot cost‐effectively realize the high‐throughput fabrication of FEEGs, thereby calling for a novel construction method capable of large‐scale production of FEEGs with asymmetric structures. Furthermore, current methods for serial and parallel connections of FEEGs rely on additional current collectors or metal‐based interconnectors,^[^ ^17^, ^18^, ^19^ ^]^ which also hinder the implementation of scalable integration of FEEGs. Therefore, in order to achieve large‐scale integration of FEEGs, the design and manufacturing strategies also need to realize the innovation for serial and parallel connections of FEEGs.

Screen printing, as a facile and cost‐effective manufacturing technology, has been used for the large‐scale production of flexible electronic devices such as batteries,^[^ ^20^, ^21^ ^]^ supercapacitors,^[^ ^22^, ^23^ ^]^ sensors,^[^ ^24^, ^25^ ^]^ and moisture‐enabled electric generators.^[^ ^26^, ^27^ ^]^ Specifically, the screen‐printing process begins with customizing functional ink formulations according to the performance requirements of the electronic devices. These functional inks are then sequentially deposited onto flexible substrates through pattern design and layer‐by‐layer printing to fabricate flexible electronic devices.^[^ ^28^, ^29^, ^30^ ^]^ Therefore, relying on custom‐formulated functional inks, different functional parts of the devices can be achieved on a large scale via screen printing. For instance, screen printing can be employed to realize the scalable fabrication of gel electrolyte layers of the flexible aqueous sodium‐ion batteries,^[^ ^31^ ^]^ electrode layers of the asymmetric flexible supercapacitors,^[^ ^32^ ^]^ and current collectors as well as interconnectors of the flexible supercapacitor arrays.^[^ ^33^ ^]^ These studies provide novel ideas for the mass production of hygroscopic hydrogel‐based portable water storage units, high‐throughput fabrication of asymmetric structures, and scalable preparation of current collectors and interconnectors, and also pave the way for large‐scale integrated production of FEEGs.

Herein, we demonstrate a novel manufacturing process for fully‐printed flexible evaporation‐driven electricity generators (PFEEGs) with high output power density, small size, and outstanding integrability as a practical and long‐lasting green energy supply solution for portable and wearable electronics for the first time. By adjusting the mass ratio of GO and activated carbon (AC), GO_H_ ink with high oxygen‐containing groups content and GO_L_ ink with low oxygen‐containing groups content are formulated. Subsequently, the negative electrode ink is prepared by mixing the active component of iron (Fe) with the commercial conductive carbon (C) ink. In contrast, the commercial conductive C ink is directly used as the positive electrode ink. Additionally, lithium chloride/polyvinyl alcohol (LiCl/PVA) hydrogel inks with excellent hygroscopicity are also prepared. The fabrication of PFEEGs relies on patternable, cost‐effective, scalable, layer‐by‐layer printing of the above custom‐formulated functional inks to sequentially form GO asymmetric structures, current collectors, and hygroscopic water storage units. The GO asymmetric structure constructed by screen printing can increase the proton concentration gradient within the PFEEG. The hygroscopic unit continuously absorbs moisture from the surrounding environment to form the water source while simultaneously evaporating moisture through the non‐hygroscopic part, establishing a sustained capillary flow circulation. The capillary flow further promotes the directional migration of ions generated from the redox reaction of the negative electrode, thereby enhancing the ion current density and enabling the PFEEG to achieve high electric output performance sustainably. Consequently, a PFEEG unit (0.5 cm × 1 cm × 38 µm) can produce a voltage output of ≈0.8 V at relative humidity (RH, 20–90%) and offer a maximum power density of 1.55 µW cm^−2^ at 70% RH. With the advantages of the flexible pattern design of the screen plate, hundreds of PFEEGs connected in series or parallel can be easily printed on the flexible substrate. Up to 200 PFEEG arrays in series or parallel can provide a voltage or current output of 152.41 V or 1.02 mA. The as‐prepared PFEEG arrays not only eliminate the need for additional current collectors or metal‐based interconnectors, but also present extraordinary mechanical flexibility and linearly scalable electric output performance, catering to the energy supply demands of commercial electronic devices. Furthermore, a printed flexible circuit based on PFEEG arrays is fabricated on various substrates, demonstrating the enormous potential of screen‐printing technology and the prepared PFEEG arrays in flexible electronics. In addition, a fully integrated self‐powered sensing system with the PFEEG arrays, a humidity sensor and light‐emitting diode (LED) arrays is fabricated on a flexible substrate. This integrated system is successfully applied to visually monitor human breathing rate or fruit damage levels, demonstrating the application potential of PFEEGs in portable and wearable integrated electronics. This work presents a low‐cost and high‐throughput fabrication method to efficiently achieve the construction of asymmetric structures and the scalable integration of FEEGs. It provides novel insights into the applications of FEEGs in flexible electronics.

## 2 Results and Discussion

### 2.1 Fabrication of the PFEEG

**Figure 1:**
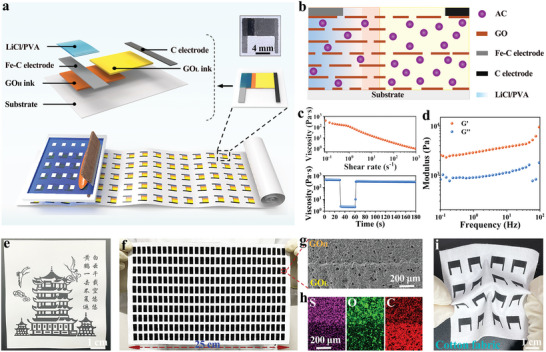
Structure and characterization of PFEEGs. a) Schematic illustration of large‐scale fabrication of PFEEGs, and the structure as well as the actual photo (inset) of a PFEEG unit. b) Schematic illustration of components distribution of the PFEEG. c) Rheological properties of GO_H_ inks with viscosity plotted as a function of shear rate (top) and interval shearing time (bottom, alternating the shear rate between 0.1 and 200 s^−1^ to simulate the extrusion process). d) Modulus (G’ and G’’) of the GO_H_ ink versus frequency. e) The photo of a pattern of the Yellow Crane Tower printed with the GO_H_ ink on the copy paper substrate. f) The photo of 300 PFEEGs (without electrodes) printed on the copy paper substrate (15 cm × 25 cm). g) SEM image and h) EDS element maps of sulfur, oxygen, and carbon at the interface of GO_H_ and GO_L_. i) Photo of PFEEG arrays printed on a cotton fabric substrate.

**Figure** **1**a shows the large‐scale manufacturing of PFEEGs on a flexible substrate through screen printing. The PFEEG unit consists of GO_H_, GO_L_, asymmetric electrodes (Fe─C and C electrodes), and LiCl/PVA. The screen printing of a PFEEG depends on the customized formulation of 5 functional inks, corresponding to the 5 parts of the PFEEG, namely GO_H_, GO_L_, Fe─C electrode (negative electrode), C electrode (positive electrode), and LiCl/PVA. It should be noted that the rheological properties of the ink can play a crucial role in the screen‐printing process. The ink should exhibit typical shear thinning behavior (high viscosity and shear thinning behavior) to ensure that it can be continuously and rapidly squeezed through the screen mesh during the screen‐printing process and can be quickly solidified when deposited on the substrate, resulting in a high‐resolution pattern. The GO_H_ ink is composed of GO, AC, sodium dodecyl benzene sulfonate (SDBS), and carboxymethyl cellulose (CMC) aqueous solution (with a mass ratio of 3:12 for GO and AC). The introduction of CMC as a colloidal binder not only imparts excellent wettability to the GO_H_ layer, facilitating the movement of capillary water flow within GO_H_, but also allows for the viscosity modulation of the ink system, rendering it suitable for screen printing.^[^ ^34^, ^35^ ^]^ As a surfactant, SDBS can reduce surface energy and is water soluble.^[^ ^36^, ^37^ ^]^ The contact angle of the GO_H_ ink on the copy paper substrate is 83.1°, demonstrating that the GO_H_ ink has good wettability on the substrate (Figure S1, Supporting Information). As shown in Figure 1c, the rheological properties of the GO_H_ ink are tested at shear rates ranging from 10^−1^ to 10^3^ s^−1^. The GO_H_ ink exhibits significant shear thinning behavior (viscosity of ≈4.5 × 10^2^ Pa·s), indicating its ability to be continuously extruded through the screen mesh and then rapidly solidify.^[^ ^22^ ^]^ Furthermore, Figure 1d shows the storage modulus (G’) and loss modulus (G’’) of the GO_H_ ink as a function of frequency. As the frequency varies from 10^−1^ to 10^2^ Hz, the G’ of the GO_H_ ink surpasses the G’’, indicating that the GO_H_ ink has a desirable viscoelastic property and can maintain long‐term dispersion stability in a static state.^[^ ^38^ ^]^ The above results demonstrate the outstanding printability of the GO_H_ ink. Therefore, it can be used to screen print large‐area patterns on the copy paper substrate, such as the Yellow Crane Tower in Figure 1e. The GO_L_ ink consists of GO, AC, and CMC aqueous solutions (with a mass ratio of 1:14 for GO and AC). Similarly, rheological test results for the GO_L_ ink indicate its desirable printability, enabling the large‐area printing patterns on the copy paper substrate (Figures S2 and S3, Supporting Information). The electrical conductivity and the relative content of oxygen‐containing groups in the inks can be altered by modulating the mass ratio of GO and C in both GO_H_ and GO_L_ inks. The commercial conductive C ink is directly employed as the C electrode ink. Meanwhile, the Fe─C electrode ink is prepared by uniformly mixing commercial C ink with the Fe powder. The LiCl/PVA ink is prepared by mixing LiCl aqueous solution with PVA aqueous solution, and the introduction of PVA promotes the gelation of the ink, facilitating ink printing and the formation of a hygroscopic film after drying. According to rheological test results, all three inks (2 electrodes and LiCl/PVA inks) exhibit desirable printability (Figure S4, Supporting Information). The detailed process of fabricating PFEEGs by screen printing is shown in Figure S5 (Supporting Information), with the copy paper selected as a flexible substrate. Scanning electron microscopy (SEM) and atomic force microscopy (AFM) images of the surface of the copy paper shows that there is a micro/nanostructure composed of lots of interwoven fibers on the surface of the copy paper, which is beneficial to ink deposition (Figure S6, Supporting Information).^[^ ^39^ ^]^ First, distinct screen patterns for different parts of PFEEGs are designed based on the structural composition. Inspired by the utilization of graphene oxide to construct an asymmetric structure enhancing the power generation of PFEEGs,^[^ ^15^, ^16^ ^]^ and leveraging the advantages of layer‐by‐layer screen printing,^[^ ^20^ ^]^ GO_H_ and GO_L_ are sequentially printed onto the copy paper substrate. The above process facilitated the straightforward construction of an asymmetric structure with the oxygen‐containing groups gradient. Moreover, the GO_H_ exhibits better hydrophilicity (Figure S7, Supporting Information). As shown in Figure S8 (Supporting Information), SEM and AFM images of the printed GO_H_ and GO_L_ layers reveal the deposition of functional materials on the surface of the copy paper substrate, forming abundant micro/nanochannels conducive to forming capillary water flow.^[^ ^5^ ^]^ Subsequently, the Fe─C electrode and C electrode are screen‐printed on the left side of the GO_H_ and the right side of the GO_L_, respectively. Inspired by the enhancement of salt solution evaporation‐driven electricity generation in original battery reactions, the Fe─C electrode and C electrode are selected as the negative and positive electrodes, respectively. The redox reaction of the electrodes can continuously supply metal cations for the negative electrode region.^[^ ^18^, ^40^, ^41^ ^]^ Finally, LiCl/PVA is printed on the side of the Fe─C electrode, enabling the spontaneous absorption of water molecules from the surrounding environment, thereby providing a sustainable water source for the negative electrode region of the PFEEG.^[^ ^37^, ^42^ ^]^ The schematic illustration of the internal component distribution in the PFEEG with an asymmetric structure is shown in Figure 1b. By following these steps, PFEEGs can be manufactured without the need for additional electrode assembly and fixation procedures. These results indicate that screen printing is a facile and scalable approach for manufacturing FEEGs. This screen‐printing strategy also enables the full integration of PFEEGs with functional devices (LED arrays, humidity sensors), which will be discussed in the later section.

As shown in Figure 1f, 300 PFEEGs of asymmetric GO_H_‐GO_L_ structures (without electrodes) are readily manufactured by screen printing on a copy paper substrate (15 cm × 25 cm). Fourier transform infrared (FTIR) spectra, X‐ray photoelectron spectroscopy (XPS), and Raman spectra of the GO_H_ and GO_L_ regions indicate the successful construction of the asymmetric structure (Figure S9–S11, Supporting Information). The SEM image displays the close connection at the interface of GO_H_ and GO_L_ (Figure 1g). Moreover, as demonstrated from energy‐dispersive X‐ray spectroscopy (EDS) mapping images (Figure 1h), there is an asymmetric distribution of oxygen‐containing groups along the planar direction. In addition, as shown in Figure 1i, PFEEGs can also be manufactured onto a cotton fabric substrate through screen printing, showcasing excellent mechanical flexibility.

### 2.2 Electric Output Performance of the PFEEG

**Figure 2:**
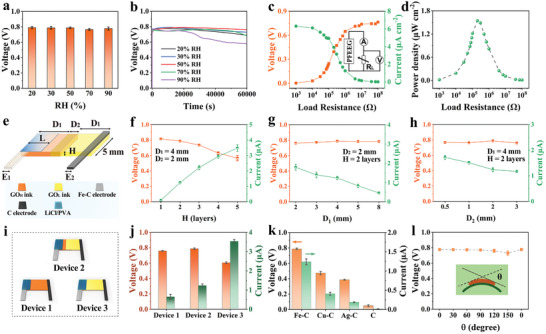
Electric output performance of the PFEEG. a) Voltage and b) continuous voltage output of the PFEEG under various RH conditions. c) Electric output of the PFEEG with external resistances changed from 10^3^ to 10^8^ Ω (70% RH). The inset presents a schematic of the equivalent circuit of the test. d) Output power density of the PFEEG calculated under various resistance loads. e) Schematic structure of a PFEEG. Electric output of PFEEGs with different f) H, g) D_1_, and h) D_2_. i) Schematic of the PFEEGs with different printed parts. Electric output of j) Device 1, 2 as well as 3, and k) the PFEEG with different negative electrodes (Fe─C, Cu─C, Ag─C, and C). l) Voltage of the PFEEG under different bending angles. The inset is a schematic of the PFEEG under the bending state.

To investigate the influence of humidity on the power output of the PFEEG, long‐term electricity generation of the PFEEG is tested under various humidity conditions. As shown in **Figure** **2**a,b, the voltage output of the PFEEG with a small size (0.5 cm × 1 cm × 38 µm) can be maintained stably at ≈0.80 V for a long time over a wide humidity range (20‐90% RH) (Figure S12, Supporting Information). Notably, the PFEEG exhibits an optimal voltage output performance at 50% RH. Even under extremely high humidity conditions (90% RH), the PFEEG can still reach 0.58 V after a continuous voltage output of up to 60 000 s, which indicates that the PFEEG has excellent humidity adaptability. Furthermore, with humidity increasing from 20% to 90% RH, the current output of the PFEEG steadily increases from ≈0.52 to 5.24 µA (Figure S13, Supporting Information), and it can reach a peak output current of ≈2.76 mA cm^−3^ (90% RH), attributed to enhanced moisture absorption and charge transfer. The electric output performance of the PFEEG cannot be achieved only by the redox reaction of the negative electrode (Figure S14, Supporting Information).^[^ ^41^ ^]^ Furthermore, due to the superb hygroscopic capability of the LiCl/PVA, the PFEEG can still repeatedly generate desirable electricity after drying cycles (Figure S15, Supporting Information). As shown in Figure 2c, the power output performance of the PFEEG under different external resistances is further investigated. As the external resistance changes from 10^3^ Ω to 10^8^ Ω, the voltage of the PFEEG increases, and the current gradually decreases. When the external resistance is 2 × 10^5^ Ω, the PFEEG can achieve a maximum power density of 1.55 µW cm^−2^ (Figure 2d), surpassing the power density of most reported FEEGs (Table S1, Supporting Information).

As the PFEEG is fabricated through a layer‐by‐layer screen‐printing process, the size of each part plays a crucial role in the power generation performance of PFEEGs. The schematic structure and design parameters of the PFEEG are illustrated in Figure 2e. The thicknesses of GO_H_ and GO_L_ are controlled by the number of screen‐printing layers (H), and they are generally proportional to H (Figure S16, Supporting Information). When H increases from 1 to 5 layers, the voltage values and retention time gradually decrease, while the current gradually increases (Figure 2f; Figure S17, Supporting Information). The possible explanation is that the increase in printed thickness reduces the resistance of the device, but the aggregation of AC and GO in the PFEEG increases, resulting in the decrease in the effective contact area between AC and GO and the water molecules. These results lead to fewer diffused ions and lower charge transport resistance.^[^ ^8^, ^11^, ^17^ ^]^ When H of GO_H_ and GO_L_ are set at 2 layers, the PFEEG exhibits optimal output performance, and the average thickness of GO_H_ and GO_L_ is ≈38 µm, considered the optimal thickness for the PFEEG. The distance from GO_H_ and GO_L_ to electrodes (D_1_) is a crucial parameter for the power output performance, as illustrated in Figure 2g. As D_1_ increases from 2 to 8 mm, the current output consistently decreases while the voltage output remains relatively constant, and the retention time prolongs (Figure S18, Supporting Information). And it is attributed to the increased distance of cation transport from the negative electrode to the positive electrode, resulting in an increased barrier to ion transfer. Considering the significance of printing precision and size for large‐scale integration of PFEEGs, 4 mm is selected as the optimized D_1_. Moreover, the overlap width (D_2_) of GO_H_ and GO_L_ can also influence the power output performance. The presence of D_2_ ensures good contact between GO_H_ and GO_L_. When D_2_ increases from 0.5 to 3 mm, the current output gradually decreases, while the voltage output remains nearly unchanged, and the retention time increases gradually (Figure 2h; Figure S19, Supporting Information). Similarly, the increase in D_2_ would also increase the ion transport distance. Therefore, 2 mm is selected as the optimized overlap width. Ultimately, the optimized length of the PFEEG is 1 cm. Besides, the influence of the overlap widths of the negative and positive electrodes with GO_H_ and GO_L_ (E_1_ and E_2_), LiCl/PVA width (L) and LiCl content on the power output performance are also investigated (Figures S20–S22, Supporting Information). In addition, as the PFEEG's width (W) increases, the voltage output remains stable, while the current output increases (Figure S23, Supporting Information). After optimization, E_1_, E_2_, L, and W are set to 1.5, 1, 3, and 5 mm, respectively. The optimized mass ratio of LiCl and PVA aqueous solution is 1:1.

The influence of the asymmetric structure of the PFEEG on the electric output performance is further investigated. The PFEEG with the above‐optimized size (0.5 cm × 1 cm × 38 µm) and containing the GO_H_‐GO_L_ asymmetric structure is named Device 2. In comparison, the PFEEG with the same size but consisting only of GO_H_ or GO_L_ are named Device 1 and Device 3, respectively (Figure 2i). In Device 1, Device 2, and Device 3, the content of AC increases sequentially, while the content of GO decreases sequentially. The resistances of these three devices (without LiCl/PVA) decrease in order, exhibiting robust Ohmic contact behavior (Figure S24a, Supporting Information).^[^ ^11^, ^17^ ^]^ Furthermore, the electrochemical impedance results of Device 1, 2, and 3 indicate a sequential increase in ionic conductivity of these three structures (Figure S24b, Supporting Information).^[^ ^42^ ^]^ As the resistances of Device 1, Device 2, and Device 3 decrease gradually, the voltage output first shows no significant increase. Then it decreases significantly, accompanied by a shorter duration, while the current output increases (Figure 2j and Figure S24c, Supporting Information). It is attributed to the decreasing device resistance, resulting in a reduced barrier to ion transport.^[^ ^8^, ^17^ ^]^ The above results indicate that the facile construction of the GO_H_‐GO_L_ asymmetric structure via screen printing can significantly enhance the electric output performance of the PFEEG. As shown in Figure 2k, when the negative electrode of PFEEG is inert (C electrode), the device exhibits faint electric output performance. It is attributed to the high ion concentration in the moisture‐absorbing layer (LiCl/PVA), which reduces the Debye length and weakens the overlap of the EDL formed in the micro/nanochannels.^[^ ^41^, ^43^ ^]^ In addition, by introducing different active metal components to construct the negative electrode, the output performance of the PFEEG can also be enhanced and the performance improvement is correlated with the metal activity.^[^ ^41^ ^]^ The mechanical flexibility of the PFEEG is tested, as shown in Figure 2l. When the bending angle of the PFEEG increases from 0° to 150° and finally returns to 0°, the electric output performance of the PFEEG remains nearly unchanged. The performance can also maintain stability following the 300 cycles of bending tests at a 150° bending angle, further demonstrating the excellent mechanical robustness of the PFEEG (Figure S25, Supporting Information).

To investigate the advantages of the planar structure design on the PFEEG's electric output performance, a standard sandwich‐structured PFEEG is fabricated for comparative analysis against the planar‐structured PFEEG. The schematic illustration of a standard sandwich‐structured PFEEG is presented in Figure S26a (Supporting Information). The size parameters of each part of the PFEEG of sandwich structure are shown in Figure S26b (Supporting Information), and the area of the sandwich‐structured PFEEG is 1 cm^2^. To prevent direct edge contact between each part that could cause a short circuit between the positive and negative electrodes, the GO_L_ layer is slightly larger than that of the C electrode, and one end of the GO_H_ layer extends outwards. This design can prevent the region where the Fe─C electrode extends from directly contacting other parts of the sandwich structure beneath it. The output voltage of the sandwich‐structured PFEEG is ≈10.8 mV, and the output current is ≈17.9 µA. Both the voltage and current outputs cannot be maintained stably over a long time. Although the current output of the sandwich‐structured PFEEG is several times larger than that of the planar‐structured PFEEG, its voltage output is much smaller (Figure S26c–e, Supporting Information). The reason is that both GO_H_ and GO_L_ layers exhibit good hydrophilicity (Figure S7, Supporting Information). When the top layer (LiCl/PVA gel) of the sandwich‐structured PFEEG absorbs moisture, water can easily permeate from GO_H_ to GO_L_ and finally to the C electrode, causing a short circuit in the sandwich structure. Consequently, the sandwich‐structured PFEEG generates a very small voltage and a relatively larger current, but both the voltage and current outputs rapidly decay to ≈0. These results demonstrate the reasonability of the planar structure design of the PFEEG.

### 2.3 Electricity Generation Mechanism of the PFEEG

**Figure 3:**
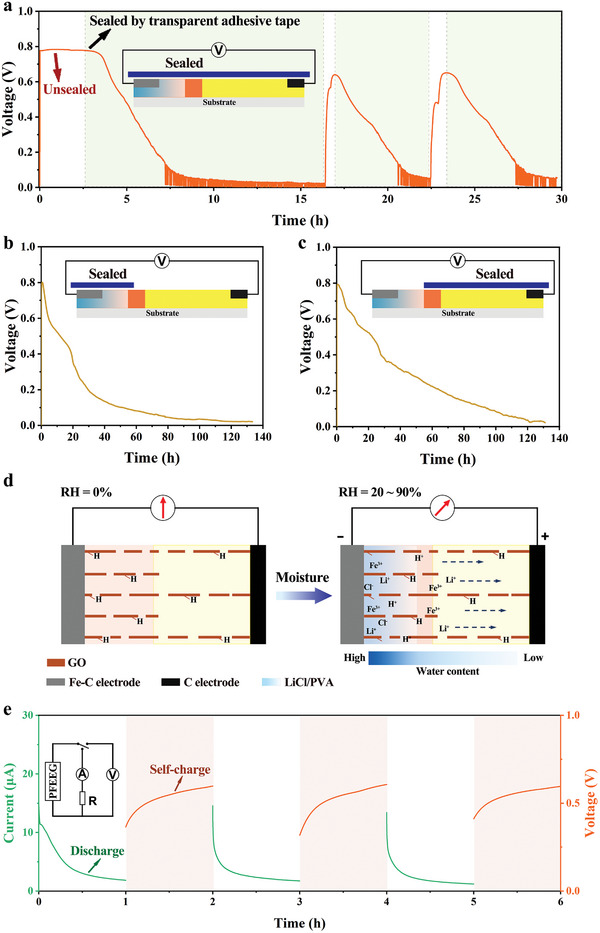
a) Voltage signal of the PFEEG when completely sealed by transparent adhesive tape and unsealed cycles. b) Voltage signal of the PFEEG with the hygroscopic part (LiCl/PVA) surface sealed. c) Voltage signal of the PFEEG with the non‐hygroscopic part surface sealed. d) Schematic diagram of the electricity generation mechanism. e) Three discharging‐charging cycles of the PFEEG with a switch and an external resistance (70% RH). The inset shows a schematic of the equivalent circuit of the test.

To understand the electricity generation mechanism of the PFEEG, the following control experiments are carried out. As shown in **Figure** **3**a, the planar PFEEG can initially continuously generate a voltage of ≈0.8 V under the condition of 50% RH. Subsequently, when the flat PFEEG is completely sealed with transparent adhesive tape, the voltage output gradually decreases to almost zero. The above unsealed and sealed processes are repeated three times, and the consistent trend in voltage output indicates that electricity generation is highly dependent on the presence of water in the environment.^[^ ^18^ ^]^ When only the hygroscopic part (LiCl/PVA) of the PFEEG is sealed, the voltage output is difficult to maintain stability and decreases rapidly (Figure 3b), demonstrating that the hygroscopic part can hardly absorb moisture through the non‐hygroscopic part. Moreover, with the decrease of water content in the hygroscopic part, the redox reaction at the electrodes becomes insufficient. However, due to the good water retention properties of the hygroscopic LiCl/PVA,^[^ ^44^ ^]^ the voltage decreased to ≈0.45 V after continuous output for ≈17 h. When only the non‐hygroscopic part of the PFEEG is sealed, the voltage is maintained at ≈0.8 V at first and then to decrease slowly after ≈3 h (Figure 3c). This initial stability in voltage output is due to the establishment of the water gradient upon sealing the non‐hygroscopic part, allowing for normal redox reaction at the electrodes. However, since water cannot evaporate through the non‐hygroscopic part, the PFEEG is gradually saturated with water absorption, attenuating the constructed water gradient. This ultimately leads to a slowdown in the directional migration of ions and a subsequent decrease in voltage output.^[^ ^37^, ^41^, ^45^ ^]^ When the test is conducted for ≈134 h, the voltage output of both the sealed hygroscopic part and the sealed non‐hygroscopic part of the PFEEG can decrease to nearly 0 V. Moreover, the water changes inside the PFEEG under the above three sealing conditions can be observed by infrared thermal (IR) images (Figure S27, Supporting Information). These results suggest that asymmetric moisture absorption and the directional capillary flow generated during water evaporation are crucial for the electricity generation of the PFEEG.

Zeta potential measurement indicates that GO_H_ and GO_L_ exhibit negative charges, attributed to the abundant oxygen‐containing groups on their surfaces. When the ionic solution flows through the channels with negative charges in the PFEEG, these channels are expected to exhibit high selectivity for cations while repelling anions (Figure S28, Supporting Information).^[^ ^17^, ^42^, ^46^ ^]^ Therefore, on the basis of the above, the following reasonable mechanism of electricity generation can be proposed (Figure 3d). When the PFEEG is placed in the ambient environment, LiCl/PVA begins to absorb ambient moisture and is partially dissociated into ions, resulting in a water gradient and ion concentration gradient inside the PFEEG containing a gradient of oxygen‐containing groups. Consequently, water and ions (including protons, lithium ions and iron ions) will flow directionally from the hygroscopic part to the non‐hygroscopic part through capillary channels (Figure S29, Supporting Information), thereby inducing power output through the evaporation‐induced potential and ion movement.^[^ ^42^, ^43^, ^47^ ^]^ With the asymmetric hygroscopic structure, the internal water gradient of the PFEEG can be sustained for a long time, inducing persistent directional capillary flow and ion movement driven by the synergistic effect of electrode redox reaction,^[^ ^40^, ^41^, ^48^ ^]^ thus enabling prolonged electricity generation of the PFEEG.

In addition, the PFEEG has specific self‐charging characteristics. As shown in Figure 3e, when connected to an external resistance, the PFEEG continuously outputs a current of ≈2 µA for 1 h. Upon disconnecting the external load, the PFEEG can naturally restore to its original power output after self‐charging for 1 h, demonstrating the potential for sustainable power generation.

### 2.4 Scaling Up of the PFEEG Arrays by Screen Printing

**Figure 4:**
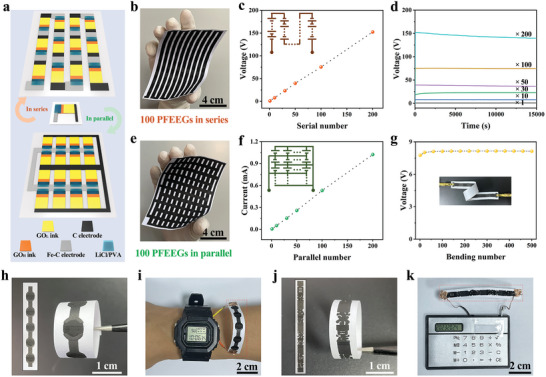
Large‐scale fabrication of PFEEG arrays by screen printing. a) Schematic diagram of the large‐scale fabrication of PFEEG arrays connected in series as well as parallel by screen‐printing technique. b) Photo of 100 PFEEG arrays in serial connection printed on the copy paper. c) The plot of voltage with different numbers of PFEEGs connected in series. The inset shows the series circuit diagram. d) The stable voltage output of PFEEG arrays with different numbers of series. e) Photo of 100 PFEEG arrays in parallel connection on the copy paper. f) The plot of current with various numbers of PFEEGs connected in parallel. The inset presents the parallel circuit diagram. g) Voltage output of ribbon‐shape PFEEGs of 10 units in serial connection during 500 bending cycles. And the inset is an actual photo. The photos of 5 circle‐shaped PFEEGs in series (h) under flat and bending (180°) states, and i) powering an electronic watch. The photos of 4 PFEEGs in series composed of 8 Chinese characters of our school motto (j) under flat and bending (180°) states, and k) powering a small commercial calculator.

Benefiting from the flexible pattern design enabled by screen printing, the electric output performance of PFEEGs can be adjusted through appropriate serial/parallel connection, facilitating the easy scaling up of the output power and effectively promoting the practical applications of PFEEGs. Due to the high conductivity and low cost of positive and negative electrodes (Figure S30, Supporting Information), the electrode inks can also serve as interconnectors for the integrated fabrication of PFEEG arrays, replacing external current collectors and metal‐based interconnectors used to connect devices. Leveraging these above features, hundreds of integrated PFEEGs utilizing serial/parallel connection can be manufactured on flexible substrates via screen printing. As shown in **Figures** **4**a and S31, (Supporting Information), the integrated PFEEGs connected in series can be fabricated by overlapping the positive electrode of each PFEEG unit with the negative electrode of the next PFEEG unit in a serpentine connection scheme. And the integrated PFEEGs connected in parallel can be achieved by connecting the positive and negative electrodes of PFEEGs, respectively, using an interdigital structure connection approach. Figure 4b presents 100 PFEEGs connected in series (7.5 cm × 14 cm) on a copy paper substrate. The voltage output of these integrated PFEEGs with a serial connection not only increases linearly with the number of devices, but also maintains stability for a long time (Figure 4c,d). Similarly, 100 PFEEGs connected in parallel (8.5 cm × 12 cm) can be easily achieved on a copy paper substrate by screen printing (Figure 4e). The current output of these PFEEGs in parallel connection also increases linearly with the increase of devices and has good output stability (Figure 4f; Figure S32, Supporting Information). These results demonstrate the excellent customizability of the electric output performance of integrated PFEEGs fabricated by screen‐printing technology. More importantly, when 200 PFEEG units are connected in series, a high voltage of 152.41 V can be generated (50% RH), and when connected in parallel, a current of 1.02 mA can be produced (90% RH). Moreover, 50 FEEGs are printed on a copy paper substrate, and their voltage values are measured to be ≈0.8 V (Figure S33, Supporting Information). The result indicates that the PFEEG has excellent robustness, which can ensure the scalability of the PFEEGs by screen printing while making these PFEEGs have almost consistent electric output performance. Furthermore, the integrated PFEEGs exhibit outstanding mechanical stability, and the output voltage of 10 PFEEGs in series can remain almost unchanged after 500 bending cycles (Figure 4g; Figure S34, Supporting Information). Therefore, a commercial thermo‐hygrometer can be powered by connecting 2 PFEEGs in series (Figure S35a, Supporting Information), and an electronic watch or a small commercial calculator can be operated by connecting 3 PFEEGs in series (Figure S35b,c, Supporting Information). The integrated 5 × 3 PFEEGs (5 series and 3 parallel connections) can also effectively power a commercial scientific calculator (Figure S35d, Supporting Information).

In addition, the screen‐printing technique is a highly effective and scalable method that enables the manufacturing of flexible and seamlessly integrated PFEEGs with designable and complex planar geometries. For instance, it is feasible to fabricate 5 circular PFEEGs connected in series (Figure 4h), which can effectively power an electronic watch, a thermo‐hygrometer, and a small commercial calculator (Figure 4i; Figure S36, Supporting Information). Moreover, it is also feasible to fabricate 4 series‐connected PFEEGs featuring our school motto composed of 8 Chinese characters (Figure 4j; Figure S37, Supporting Information), which can successfully power a small commercial calculator or a thermo‐hygrometer (Figure 4k; Figure S38, Supporting Information). For previously reported FEEGs,^[^ ^11^, ^12^, ^13^, ^18^ ^]^ the serial and parallel expansion of integrated devices requires all devices to be connected through external metal interconnectors. These integrated devices not only lack flexibility, but also occupy an ample space, severely hindering their practicality in portable and wearable electronics. Utilizing the screen‐printing technology, highly facile, low‐cost, and high‐throughput manufacturing of integrated PFEEGs with a series of parallel connections, along with customizable power output, have been achieved, offering a viable solution for the widespread applications of FEEGs.

### 2.5 Printed Flexible Circuits Powered by PFEEG Arrays

Due to the merits of large‐scale fabrication and customizable power output offered by PFEEGs, the integrated 5 × 10 PFEEGs (5 serial and 10 parallel connections) are selected as the power source unit (PSU) for subsequent studies. Using a vacuum sealer, the PSUs can be sealed in vacuum‐packed bags to isolate the devices from direct contact with the ambient environment for long‐term storage and easy portability (Figure S39a, Supporting Information). And when PSUs are needed, tear open the encapsulated vacuum‐packed bags. Furthermore, the encapsulated PSU can be placed in the ambient environment for over a month, maintaining good stability in output performance (Figure S39b,c, Supporting Information). PSUs can be conveniently attached near large electronic devices, replacing traditional batteries to provide power. To demonstrate this advantage, the PSU is attached to a commercial clock, directly driving the clock (29 cm × 33 cm) integrated with an electronic calendar and a thermo‐hygrometer (Figure S40; Movie S1, Supporting Information).

**Figure 5:**
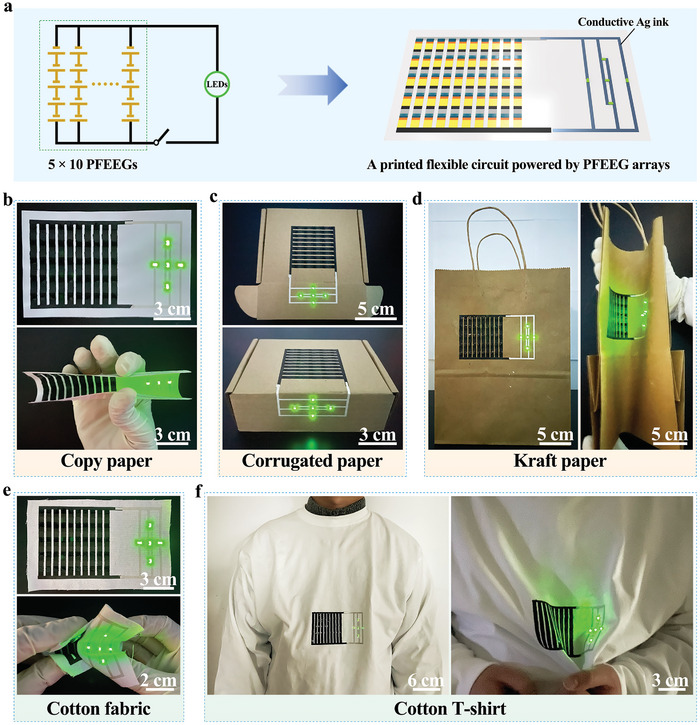
Printed flexible circuit powered by PFEEG arrays. a) Schematic illustration of the printed flexible circuit. In both flat and bent states, the integrated PFEEGs are screen‐printed on various flexible substrates, such as the b) copy paper, c) corrugated box, d) kraft paper tote bag, e) cotton fabric, and f) cotton T‐shirt, and successfully power the green LED arrays arranged in a “diamond” shape.

The fully‐printed PSU not only serves as an independent portable power module, but also can be connected seamlessly with printed flexible circuits, exhibiting tremendous application prospects in printed electronics. As shown in **Figure** **5**a, a printed integrated flexible circuit based on a PSU (5 × 10 PFEEGs) and green LED arrays is designed. The flexible circuit connecting the PSU and the LED arrays is printed with commercial conductive silver (Ag) ink, which is used to bond the green LED arrays arranged in a “diamond” shape to the circuit. Due to the excellent compatibility of PFEEGs with various substrates, the integrated flexible circuit is printed on different flexible substrates. First, the printed flexible circuit is fabricated on a copy paper substrate, where the green LED arrays can be reliably illuminated even in flat or various bent states (Figure 5b; Figure S41, Supporting Information). Moreover, in the flat state, the LED arrays can be continuously powered for over 3 h (Movie S2, Supporting Information). In addition, the printed flexible circuit is fabricated on a corrugated cardboard box and a kraft paper shopping bag. Similarly, the green LED arrays on both substrates can also be reliably illuminated even in flat or bent states (Figure 5c,d; Figures S42 and S43, Supporting Information). To investigate the application potential of the printed flexible circuit in wearable electronics, the printed flexible circuit is first fabricated on a cotton fabric substrate. Benefiting from the excellent flexibility of the cotton fabric, this printed flexible circuit can maintain excellent stability even after multiple arbitrary bending (Figure 5e; Figure S44; Movie S3, Supporting Information). Subsequently, the printed flexible circuit is fabricated on a cotton T‐shirt. When wearing this cotton T‐shirt on the body, the green LED arrays can be driven stably even as the T‐shirt undergoes deformation (Figure 5f; Figure S45, Supporting Information). Considering the biocompatibility of the PFEEGs array for wearable electronics and the safety of skin use, the PFEEGs array is directly attached to the skin surface with transparent tape for 12 h. It can be found that the PFEEGs array has no irritation to the skin and no discomfort to the human body, which proves that the PFEEGs array has great biocompatibility and safety for skin use (Figure S46a, Supporting Information). Moreover, the air permeability of the pristine fabric and the fabric‐based PFEEGs are 119.6 and 39.8 L m^−2^ s^−1^, respectively (Figure S46b,c, Supporting Information). Due to the existence of the PFEEGs array layer, the air permeability of the fabric‐based PFEEGs is lower than that of the pristine fabric. In addition, the stretchability of the pristine fabric and the fabric‐based PFEEG is shown in Figure S47 (Supporting Information). The toughness of the fabric‐based PFEEG is comparable to that of the pristine fabric, indicating that the fabric‐based PFEEG can meet the basic needs for practical use in wearable electronics. Due to the high scalability and versatility of the screen‐printing technology, it can provide a feasible approach for the printed integration of fully‐printed PSUs and multifunctional flexible electronic devices.

### 2.6 Printed Self‐Powered Visual Sensing Systems

**Figure 6:**
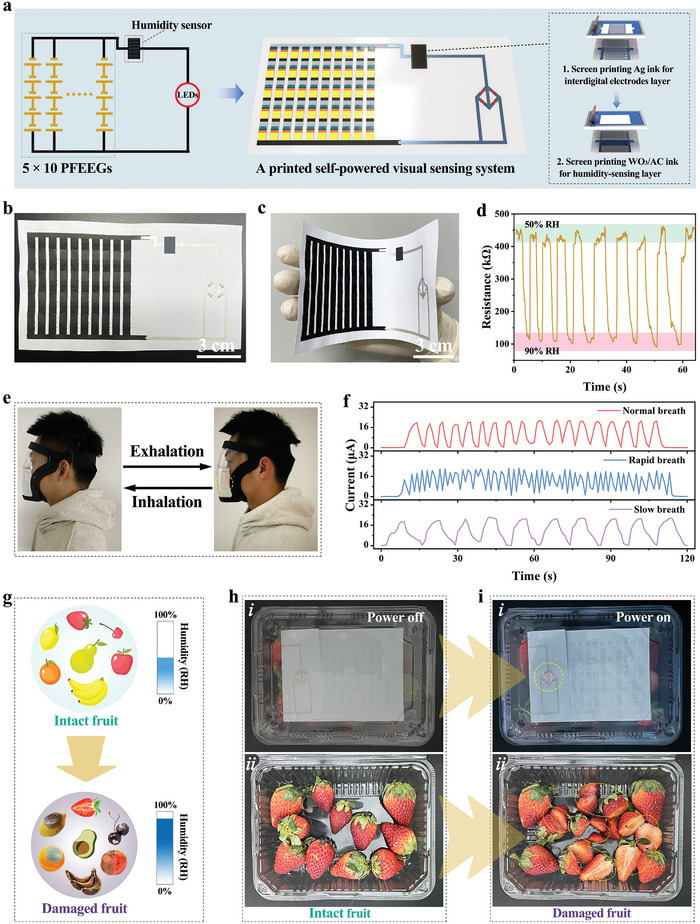
Printed self‐powered visual sensing system. a) Schematic illustration of the printed self‐powered visual sensing system. Photos of the printed system in b) flat and c) bent states. d) Repeatability of the printed flexible humidity sensor exposed at 50% and 90% RH. e) As breathing alternates between exhalation and inhalation, the red LED arrays in the visual sensing system alternate from bright to dim, visualizing the respiratory process. f) Breath monitoring signals are driven by the self‐powered sensing system in normal breathing mode (top), rapid breathing mode (middle), and slow breathing mode (bottom). g) Schematic diagram illustrating increased RH surrounding intact fruit after damage. Photos of the red LED arrays in the visual sensing system h) being off or i) being illuminated when fruit in the package box is intact or damaged.

To further demonstrate the advantages of screen‐printing technology and the unprecedented potential of PFEEGs in portable and wearable smart electronics, a fully integrated and screen‐printed flexible self‐powered visual sensing system is developed on a flexible substrate. This fully integrated system comprises the PFEEG arrays for power generation, a printed humidity sensor for signal reception, and red LED arrays for signal display. The schematic diagram of the self‐powered sensing system is shown in **Figure** **6**a. Similar to the manufacturing process of the integrated system in Figure 5a, this process begins with printing the PSU (5 × 10 PFEEGs) on a copy paper substrate. Subsequently, the conductive circuit is printed to connect a PSU, a humidity sensor, and red LED arrays. Following that, asymmetric interdigital electrodes and the WO_3_/AC humidity sensing layer are printed sequentially in the designated area for constructing the humidity sensor. Finally, the red LED arrays are bonded to the flexible circuit using commercial conductive Ag ink. By altering the RH surrounding the humidity sensor, the resistance of the sensor changes,^[^ ^24^ ^]^ resulting in a variation in the current of the red LED arrays in the flexible circuit. Consequently, the red LED arrays exhibit brightness changes, ultimately visualizing the humidity variation signal output. These processes enable the self‐powered visual sensing functionality without the need for additional rectification circuits and energy‐storage modules.

The fully integrated flexible self‐powered visual sensing system, fabricated via screen printing, measures 7 cm × 14 cm and exhibits outstanding flexibility (Figure 6b,c). As for the printed humidity sensor module, the cyclic stability between 50% and 90% RH is demonstrated for 10 cycles in Figure 6d. During the cycling process, the humidity sensor shows a stable sensing response, indicating excellent repeatability of the humidity sensor. As a demonstration for wearable applications, the self‐powered visual sensing system attached to the protective mask can monitor the human breathing rate in real‐time, and the signal can be dynamically displayed in real‐time by the flashing frequency of the red LED arrays. When exhaling, the RH around the face increases, causing the red LED arrays to light up. On the contrary, when inhaling, the RH around the face decreases, causing the red LED arrays to go off (Figure 6e). More importantly, as the respiratory rate cycles from normal to rapid and slow breathing, the self‐powered visual sensing system can consistently generate current signals in real time and stably, recording the respiratory status (Figure 6f). Additionally, the variation in inhalation frequency can be directly observed through the flashing frequency of the red LED arrays (Movie S4, Supporting Information). As a demonstration for portable applications, the self‐powered visual sensing system is attached to a fruit packaging box to monitor the preservation status of the fruit (intact or damaged) and display the result by the brightness of the red LED arrays. When the fruit in the packaging box is damaged, the RH inside the box increases, causing the red LED arrays to brighten (Figure 6g–i). Consumers can assess the status of the fruit by observing the brightness of the red LED arrays. The above potential application demonstrations serve as conceptual validations, marking the first successful construction of a fully integrated and printed flexible visual sensing system by evaporation‐induced self‐powering, consisting of an electricity generation unit, a signal reception unit, and a signal display unit. These demonstrations also inspire the integration of PFEEGs with other printable functional devices via screen‐printing technology, offering significant opportunities for widespread applications in future portable and wearable systems.

## 3 Conclusion

In summary, we develop a fully‐printed flexible evaporation‐driven electricity generator based on the asymmetric structure and the small water storage unit. By formulating inks with different functional components, the PFEEGs can be fabricated layer by layer utilizing low‐cost, scalable, and customizable screen‐printing technology. Benefiting from the GO asymmetric structure as well as the addition of Fe powder into the negative electrode, a PFEEG unit (0.5 cm × 1 cm × 38 µm) can sustainably output a remarkable voltage of ≈0.8 V at 20–90% RH and can obtain a maximum power density of 1.55 µW cm^−2^ at 70% RH. With the flexible design of screen patterns, hundreds of PFEEG arrays in serial or parallel connection can be assembled at high throughput without needing both additional current collectors and metal‐based interconnectors. 200 PFEEG arrays through a serial or parallel connection can produce a continuous power output of 152.41 V or 1.02 mA. Furthermore, by varying the number of PFEEGs connected in series or parallel, the electric output performance of the PFEEG arrays can be linearly adjusted to meet the power supply requirements of commercial electronic devices. Subsequently, PFEEGs of other shapes (e.g., circular, Chinese characters) are successfully fabricated through the pattern design and can effectively drive commercial electronic devices. To further highlight the advantages of screen printing, a printed flexible circuit integrated with PFEEG arrays and LED arrays is fabricated on different flexible substrates. The LED arrays can remain reliably illuminated when the flexible circuit is in different bending states. Subsequently, a fully integrated flexible visual sensing system based on the evaporation‐driven self‐powered supply is successfully printed. And the system includes a power generation unit, a signal receiving unit, and a signal display unit, which can realize visual monitoring of human breathing rate or fruit damage levels. In the future, by optimizing the composition of functional inks and the structure of the PFEEG, the electric output performance of PFEEGs and the duration of power generation can be further improved while promoting the development of PFEEGs to practical and commercial production. And using screen printing, precisely patterned PFEEGs can be customized to fit the geometric space of the commercial electronic device, thereby maximizing the utilization of the geometric space. Overall, this work not only provides new insights into the low‐cost, high‐throughput, and customizable integrated manufacturing of FEEGs with desirable electric output performance, but also demonstrates the extraordinary potential of PFEEGs as sustainable, clean power sources in domains such as portable and wearable electronics, as well as multifunctional printable integrated systems. And this bears tremendous implications for developing next‐generation flexible power supply devices for portable and wearable electronics.

## 4 Experimental Section

### Materials

GO was prepared according to the previous work.^[^ ^16^ ^]^ AC (YEC‐8A) was purchased from Fuzhou Yihuan Carbon Co., Ltd. (China). CMC (Mw ≈90000), SDBS (AR, 98.0%), and PVA (alcoholysis degree: 98.0–99.0 mol%) were purchased from Aladdin Chemistry Co., Ltd. Fe powder (AR, 5 µm), Cu powder (AR, 5 µm), and Ag powder (AR, 5 µm) were purchased from Qinghe County Kegong Metallurgical Materials Co., Ltd. (China). Commercial conductive C ink and Ag ink were purchased from the Taobao platform (China). LiCl was purchased from Shanghai Lingfeng Chemical Reagent Co., Ltd. (China). Absolute ethanol and ethyl cellulose (EC) were purchased from Sinopharm Chemical Reagent Co., Ltd. (China). The Deionized (DI) water (18.2 MΩ) was applied throughout the experiment.

### Preparation of Printable Functional Inks for PFEEGs

GO_H_ ink was prepared by first mixing the GO, AC, and SDBS with the mass ratio of 3:12:3 and then adding the CMC aqueous solution (5%) with a 9:25 (wt./wt.) ratio. GO_L_ ink was prepared by first mixing the GO and AC with a mass ratio of 1:14 and then adding the CMC aqueous solution (5%) with a 3:10 (wt./wt.) ratio. Fe─C electrode ink was prepared by mixing the Fe powder and conductive C ink with the mass ratio of 1:2. LiCl/PVA ink was prepared by mixing LiCl (40 wt.% in aqueous solution) and the PVA (10 wt.% in aqueous solution) with the mass ratio of 1:1. Before screen printing, all the inks were uniformly dispersed for 20 min by sonication.

### Fabrication of PFEEGs

During the screen printing, the screen plates with 200 mesh and ≈10 µm thickness were used for the GO_H_, GO_L_, and electrodes. The patterns of all screen plates were designed in advance according to the structure of a PFEEG unit or integrated arrays, including serial and/or parallel connections. The screen plates with 60 mesh and ≈20 µm thickness were used for the LiCl/PVA. To obtain the GO_H_ and GO_L_, the GO_H_ ink was screen‐printed on a flexible substrate. Then it was placed to an oven and dried at 40 °C for 20 min. Similarly, the GO_L_ ink was screen‐printed to fabricate the GO_L_. The thickness of GO_H_ and GO_L_ can be regulated by controlling the number of printing layers. To obtain the asymmetric electrodes, the Fe─C ink was screen‐printed inside the left edge of the GO_H_ and dried for 40 min (50 °C). The C ink was screen‐printed inside the right edge of the GO_L_ and dried under the same conditions as the Fe─C electrode. Then the LiCl/PVA was screen‐printed inside the left edge of GO_H_ and dried at 40 °C for 20 min. The above steps result in PFEEGs. The integrated arrays, including serial and/or parallel connections, can be fabricated through the same steps.

### Fabrication of the Flexible Circuit for Powering Green LED Arrays

First, the PSU (5 × 10 integrated PFEEG arrays with 5 in series and 10 in parallel connections) was screen‐printed on a flexible substrate. Then, the commercial Ag ink was deposited onto the flexible substrate by screen printing and dried (50 °C, 40 min) to establish the flexible circuit between green LED arrays and the PSU. The green LED arrays were welded to the connection circuit by coating the Ag ink on the contact points between green LED electrodes and the circuit, then dried at 50 °C for 20 min.

### Preparation of Printable WO_3_/AC Humidity‐Sensing Ink

WO_3_ was prepared by the previous work.^[^ ^24^ ^]^ First, the WO_3_, AC, and EC solution (5 wt.% in absolute ethanol) were mixed with a 1:1:6 (wt./wt./wt.) ratio. Then, the WO_3_/AC ink was prepared with the ultrasonication process.

### Fabrication of the Screen‐Printed Flexible Humidity Sensor

The screen plates with 200 mesh and ≈10 µm thickness were used for the interdigital electrodes and humidity‐sensing layers. First, the conductive Ag ink was screen‐printed to the corresponding position on the flexible circuit and was dried at 50 °C for 20 min to prepare the interdigital electrodes. Then, the WO_3_/AC ink was overprinted on the interdigital electrodes and was dried at 40 °C for 20 min to prepare the humidity‐sensing layer.

### Characterization and Measurements

The morphologies, Energy‐dispersive X‐ray spectra, and Raman spectrum of the samples were characterized by the SEM (Tescan MIRA). The photoelectron spectroscopy (K‐Alpha, Thermo Fisher Scientific) was used to record the XPS. FTIR spectra were obtained by a NICOLET 5700 FT‐IR Spectrometer. AFM (SPM‐9700HT) was used to investigate the surface roughness of the samples. A homemade scanning system was used to measure contact angles. A kinexus Pro+ rotary rheometer (Malvern Instruments Ltd.) was used to measure the rheological properties of functional inks. An IR camera (FLIR ETS320) was used to obtain IR images. The electric output performance measurements of the devices were performed with source meters of Keithley 2461 and DMM6500. Unless otherwise stated, these tests were conducted at ≈24 °C and 50% RH. A Testo 175‐H1 thermo‐hygrometer recorded the temperature and RH values in tests.

## Conflict of Interest

The authors declare no conflict of interest.

## Supporting information



Supporting Information

Supplemental Movie 1

Supplemental Movie 2

Supplemental Movie 3

Supplemental Movie 4

## Data Availability

The data that support the findings of this study are available from the corresponding author upon reasonable request.
