# Supplementary material for: Fully Printable Manufacturing of Miniaturized, Highly Integrated, Flexible Evaporation‐Driven Electricity Generator Arrays
Source: Adv Sci (Weinh). 2024 Dec 17;12(6):2413779. doi: 10.1002/advs.202413779 (PMC11809399; doi:10.1002/advs.202413779)
Supplement: Supplementary file 1 — Supporting Information [file ADVS-12-2413779-s001.docx]

**Fully printable manufacturing of miniaturized, highly integrated, flexible evaporation-driven electricity generator arrays**

*Qun Liu, Panwang Guo, Xinyu Zhang, Hehe Ren, Jing Liang, Quancai Li, Weinan Tang, and Wei Wu**

Laboratory of Printable Functional Materials and Printed Electronics, School of Physics and Technology, Wuhan University, Wuhan, 430072, P. R. China

**Corresponding author:** weiwu@whu.edu.cn (W. Wu)


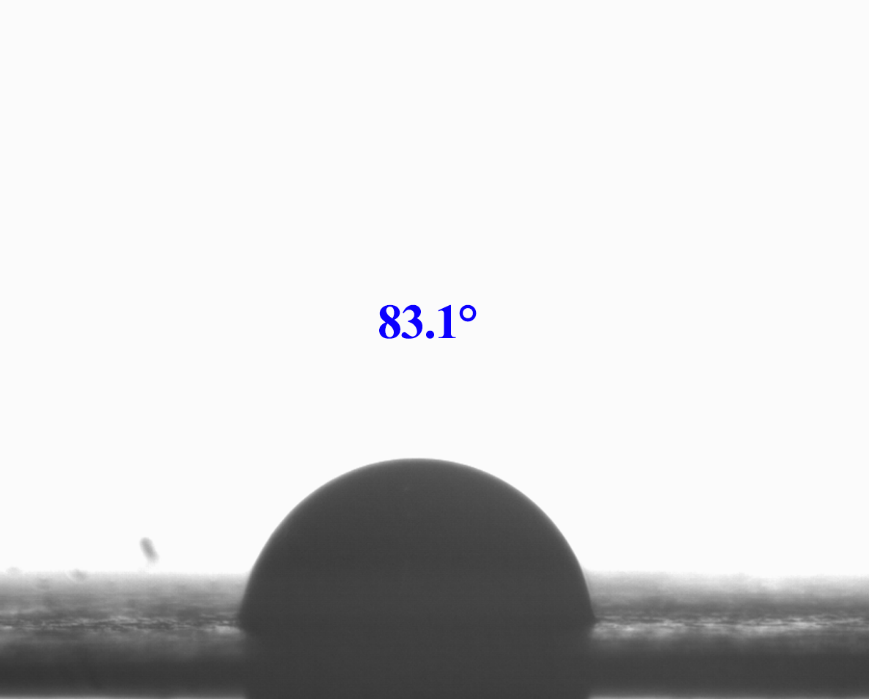


**Figure S1.** Contact angle of the GO_H_ ink on the copy paper substrate.


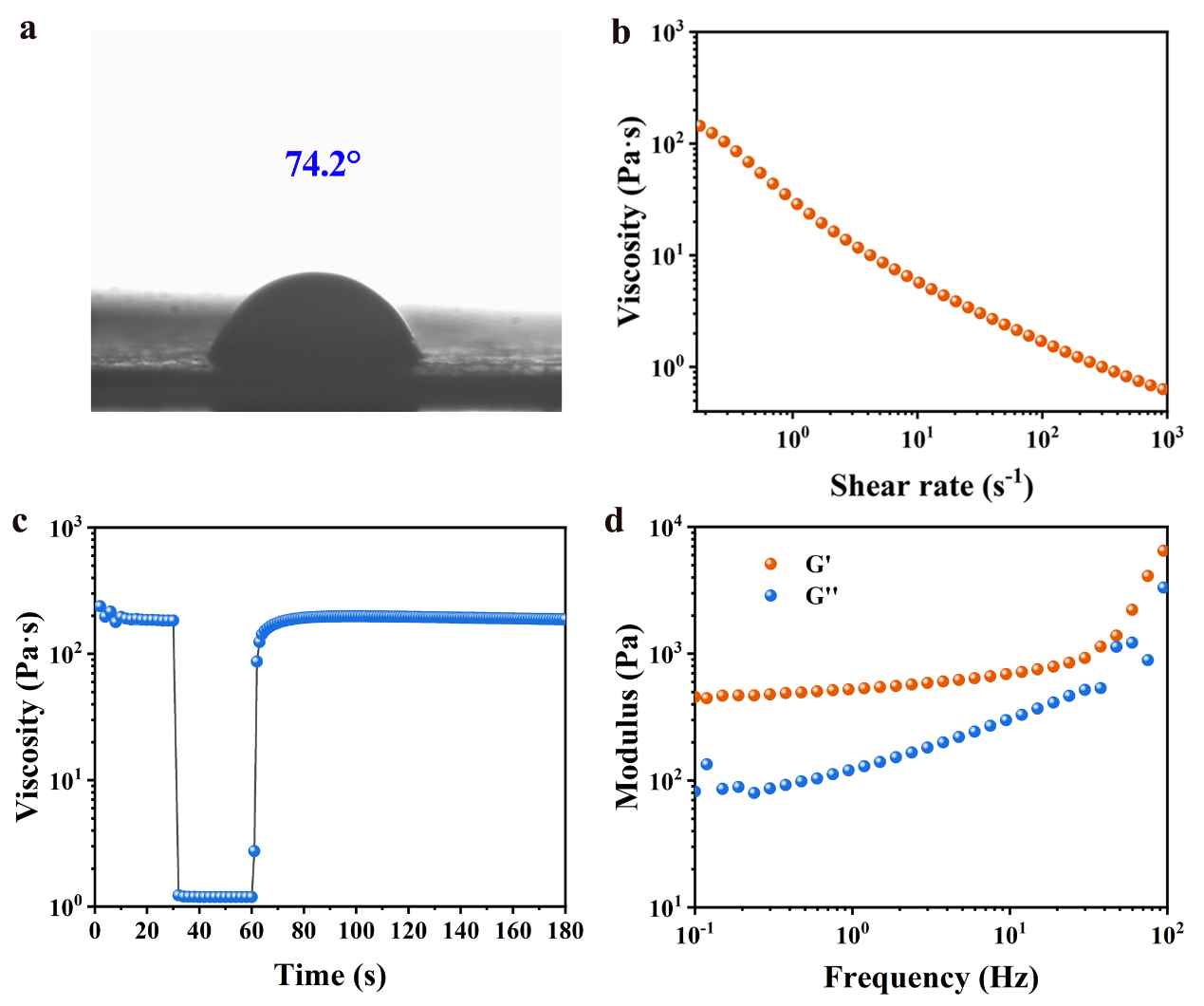


**Figure S2.** (a) The contact angle of the GO_L_ ink on the copy paper substrate. Rheological properties of GO_L_ inks with viscosity plotted as a function of (b) shear rate and (c) interval shearing time (alternating the shear rate between 0.1 s^-1^ and 200 s^-1^ to simulate the extrusion process). (d) G’ and G’’ of GO_L_ inks versus shear stress.


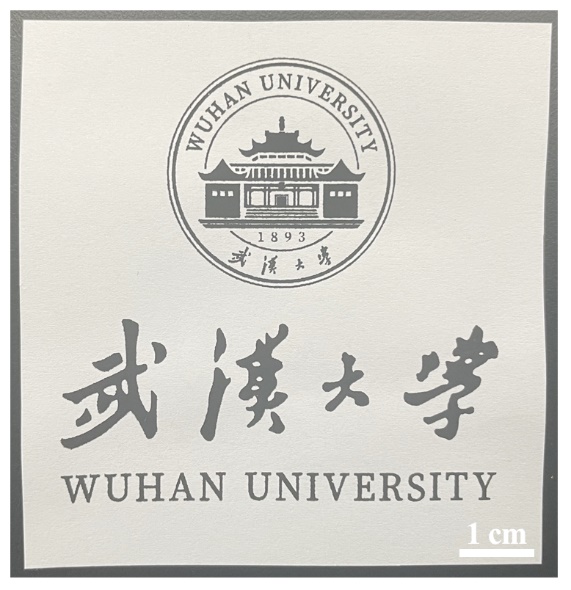


**Figure S3.** Photo of a pattern of the emblem of Wuhan University printed with the GO_L_ ink on the copy paper substrate.


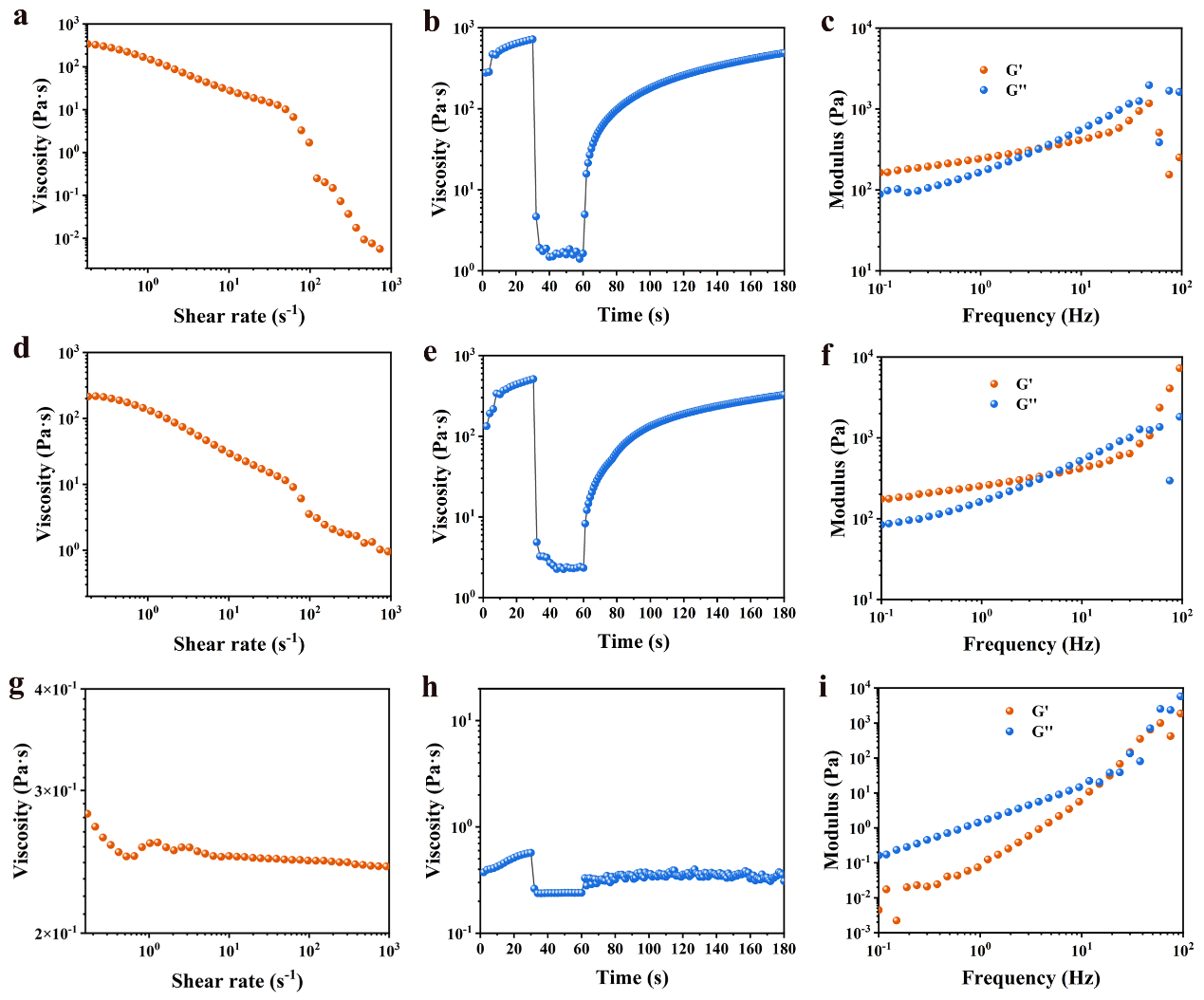


**Figure S4.** Rheological properties of Fe-C and C inks. Viscosity plotted as a function of shear rate and interval shearing time (alternating the shear rate between 0.1 s^-1^ and 200 s^-1^ to simulate the extrusion process), as well as the storage modulus (G’) and loss modulus (G’’) versus shear stress of Fe-C inks (a, b, and c), C inks (d, e, and f) and LiCl/PVA inks (g, h, and i).


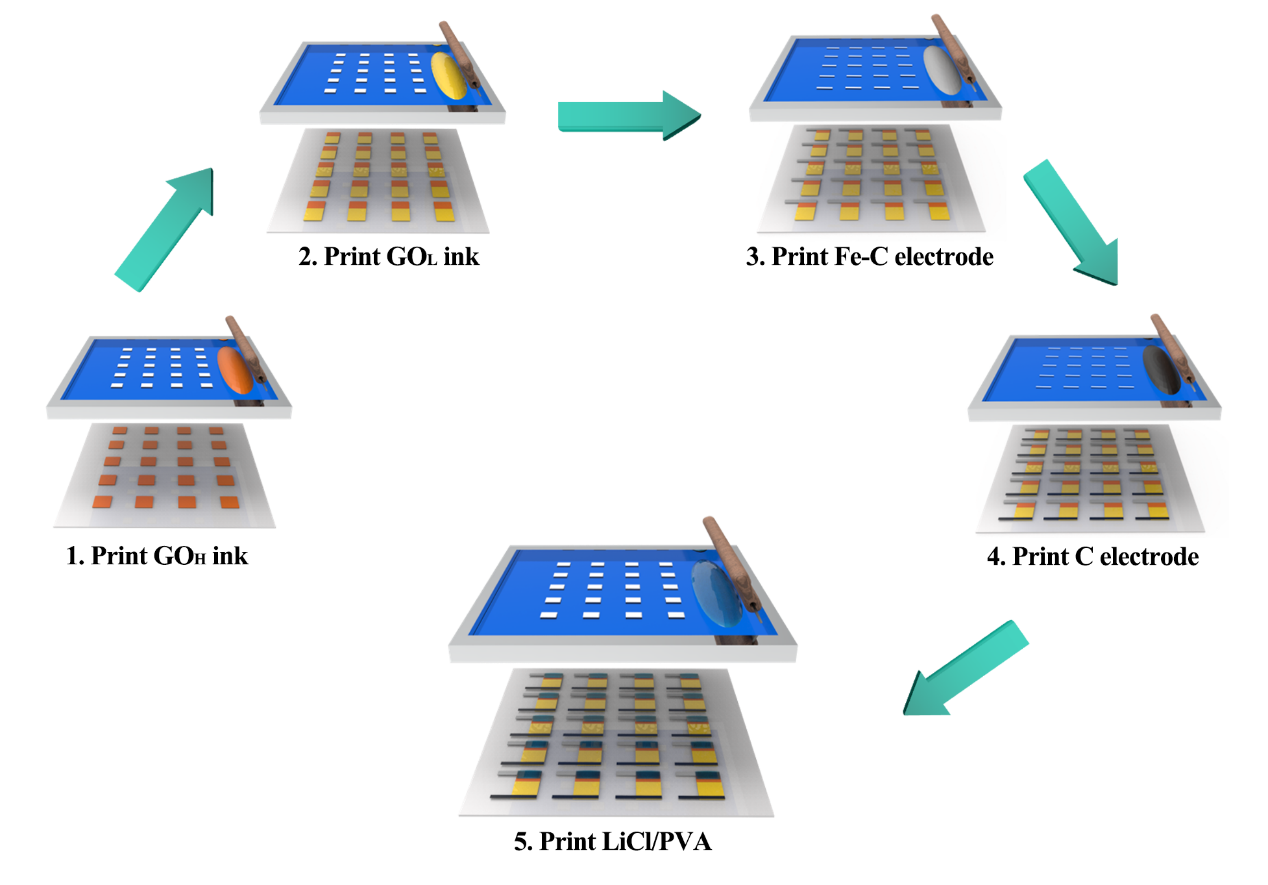


**Figure S5.** Schematic diagram of the fabrication process of PFEEGs on the copy paper substrate by screen printing.


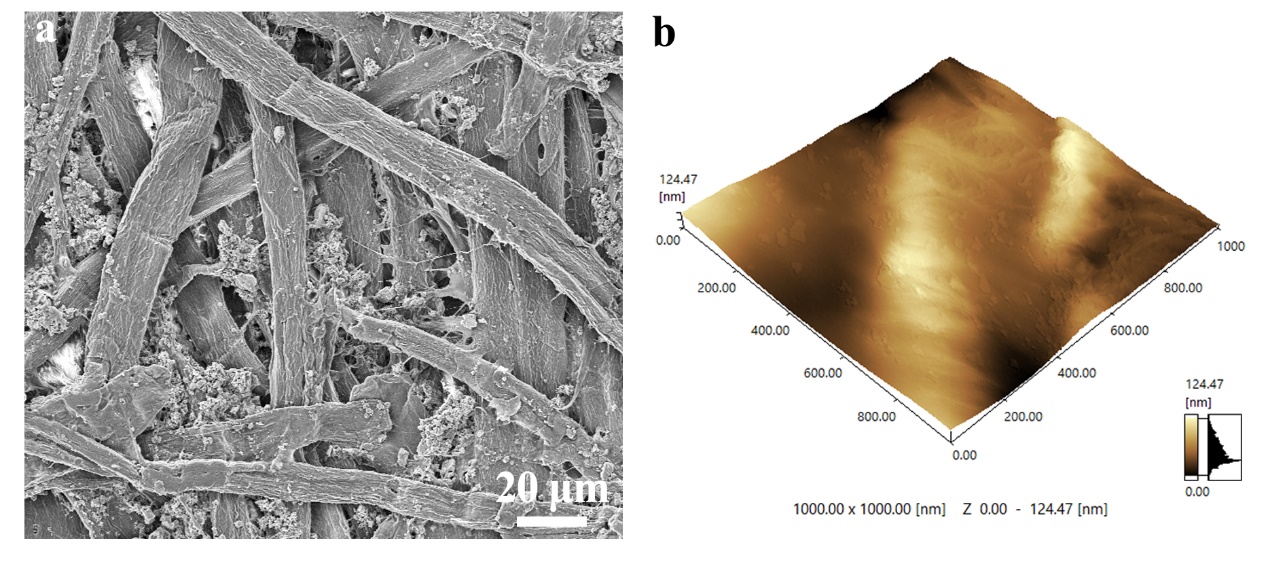


**Figure S6.** (a) SEM and (b) AFM images of the surface of the copy paper.


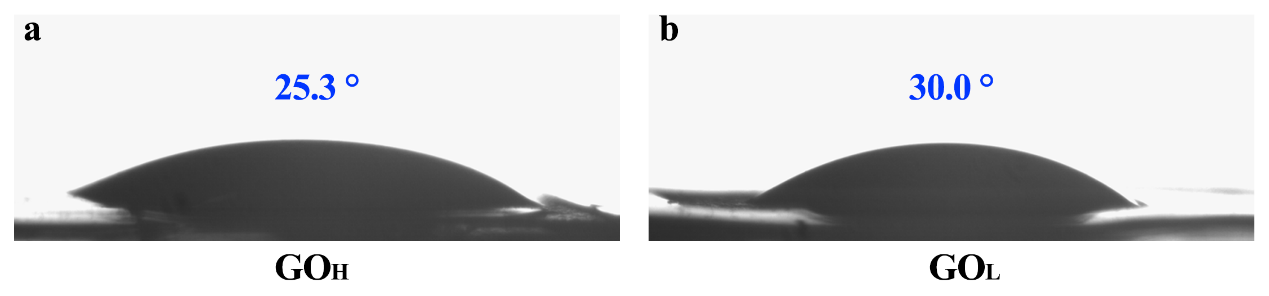


**Figure S7.** (a) Water contact angle of the GO_H_ and GO_L_.


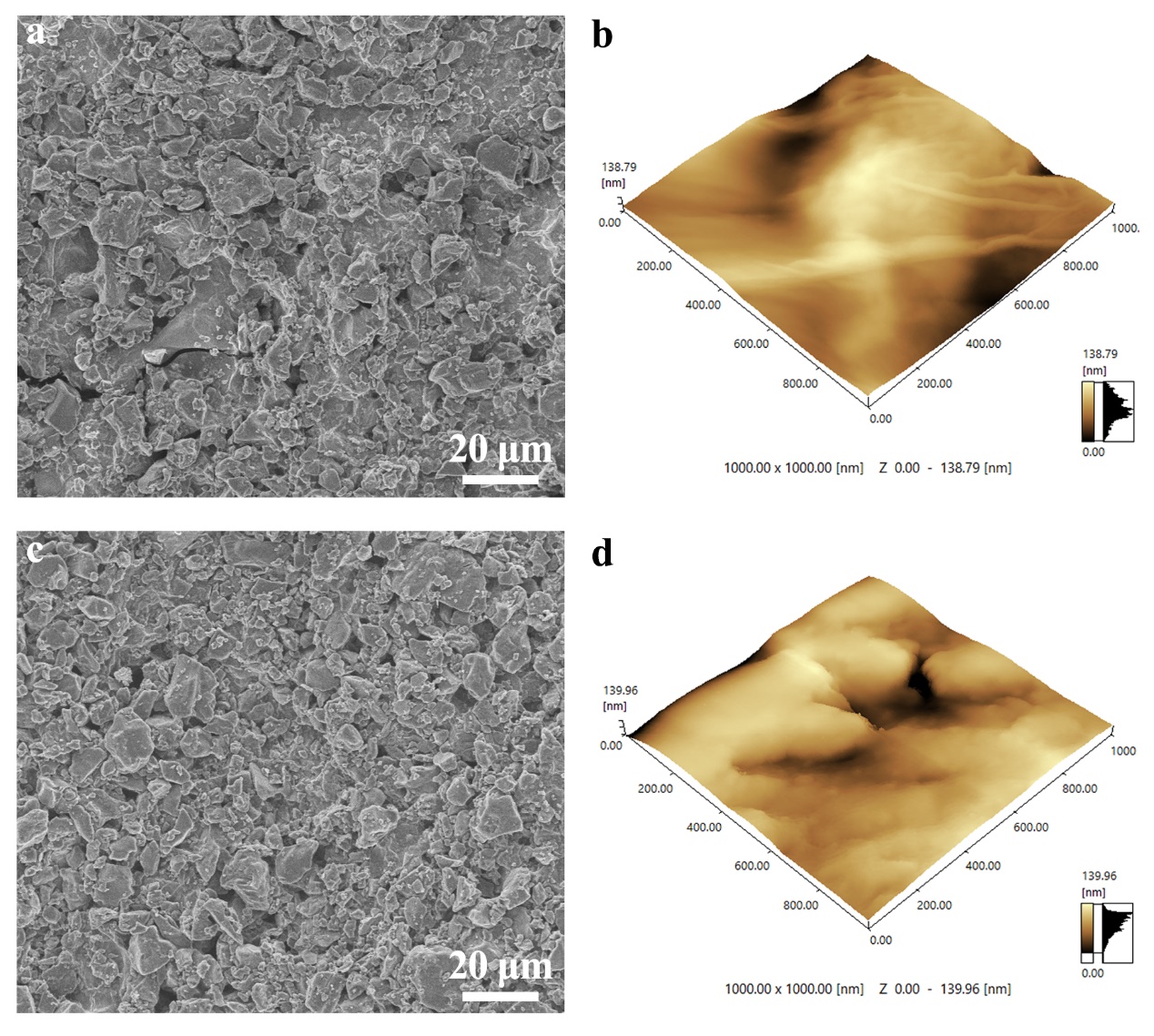


**Figure S8.** SEM and AFM images of the GO_H_ (a and b) and GO_L_ (c and d).


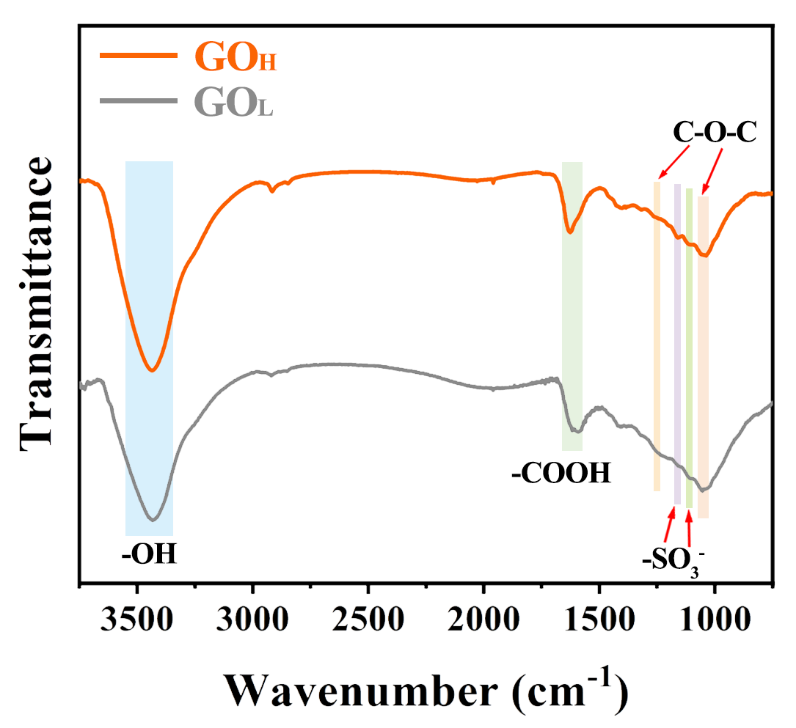


**Figure S9.** FTIR of the GO_H_ layer and the GO_L_ layer.

As shown in Figure S10, the FTIR spectrum of the GO_H_ layer shows more obvious peaks than those of the GO_L_ layer at 3436, 1625, 1260, and 1038 cm^-1^, corresponding to -OH, -COOH, and C-O-C, respectively, and 1160 cm^-1^ as well as 1038 cm^-1^, corresponding to sulfonate groups.^[1-5]^


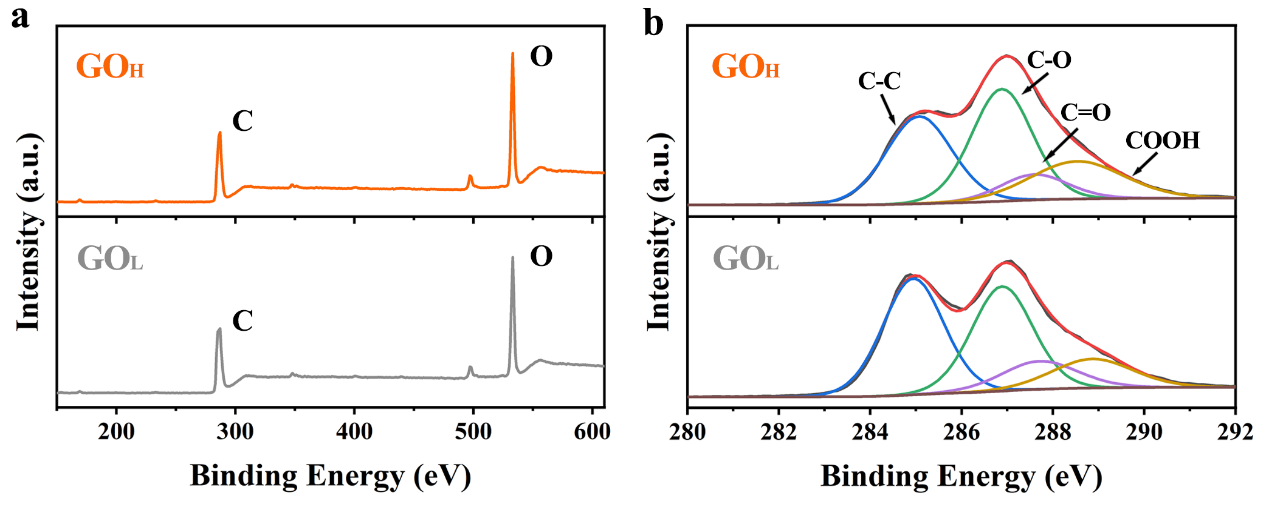


**Figure S10.** (a) XPS results of the GO_H_ layer and the GO_L_ layer. The high-resolution XPS spectrum of C1s peak of GO_H_ layer and GO_L_ layer.
As shown in Figure S11a, the XPS survey spectrum of the GO_H_ layer exhibits a predominant graphitic C 1s peak at 286 eV and O 1s peak at 532 eV,^[6-7]^ the corresponding calculated O/C ratio is about 1.63, which is much higher than that of the GO_L_ layer (~1.40). Furthermore, the high-resolution C 1s spectrum (Figure S11b) of the GO_H_ layer reveals the presence of C=C/C-C bonding (~284.8 eV), C-O bonding (hydroxyl and epoxy, ~286.6 eV), C=O (carbonyl, ~287.5 eV) and O-C=O bonding (carboxyl, ~288.7 eV). While the GO_L_ layer has relatively weaker peaks of oxygen-containing groups.^[6-7]^


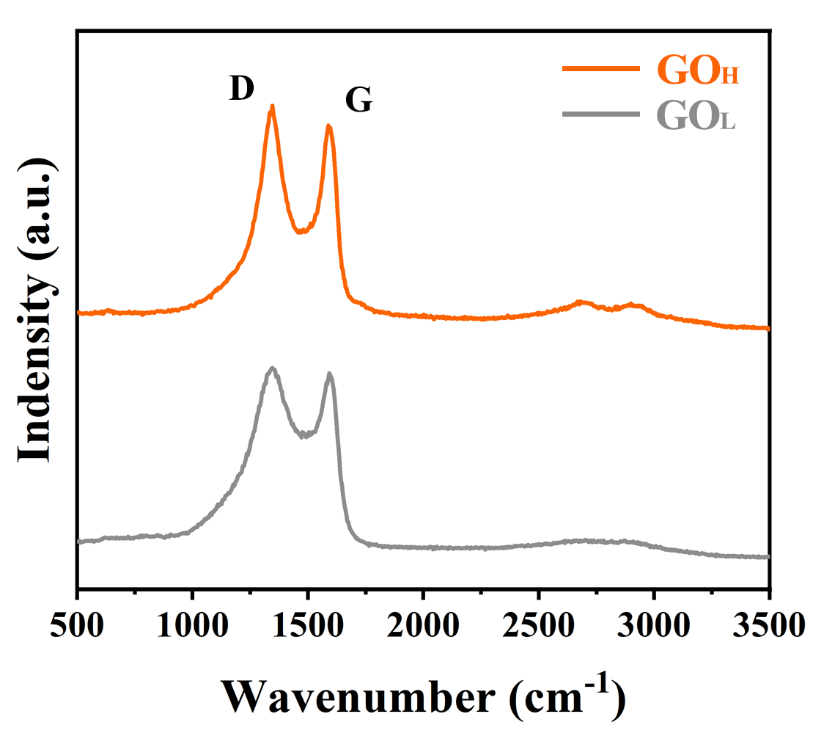


**Figure S11.** Raman spectra of GO_H_ layer and GO_L_ layer.

As shown in Figure S12, the I_D_/I_G_ (intensity ratio of D band and G band) of the GO_H_ layer is about 1.07, which is higher than that of the GO_L_ layer (~1.01), suggesting the content difference of the oxygen-containing groups.^[6]^


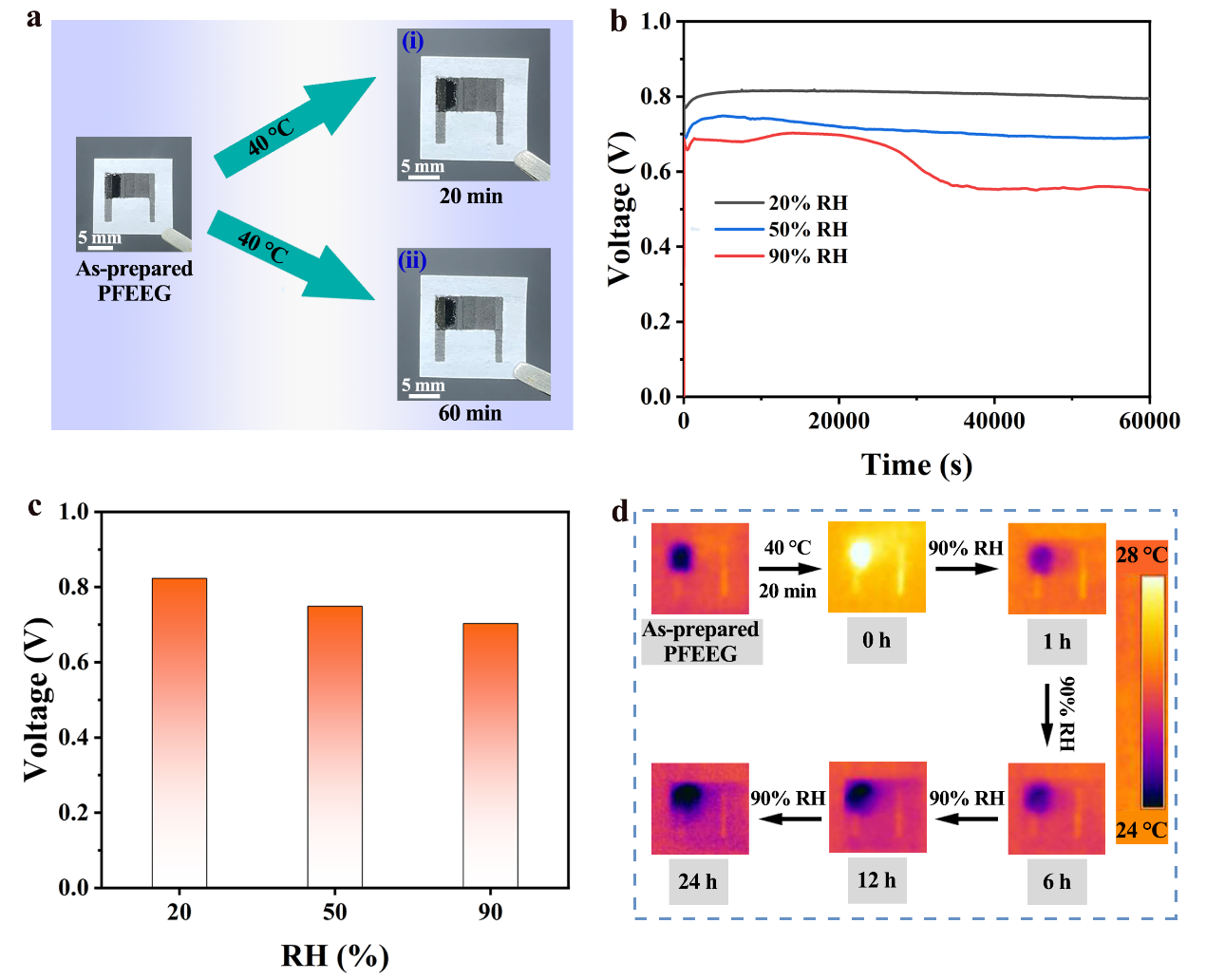


**Figure S12.** (a) Photographs of the as-prepared PFEEG and the device dried in an oven at 40 °C for (ⅰ) 20 min, and (ⅱ) 60 min, respectively. (b) Continuous voltage output and (c) voltage of the PFEEG dried at 40 °C for 60 min under the conditions of 20%, 50%, and 90% RH, respectively. (d) IR images of the as-prepared PFEEG and the device dried at 40 °C for 20 min, followed by exposure to 90% RH for 0 h, 1 h, 6 h, 12 h, and 24 h, respectively. When the device is exposed to 90% RH for 0 h, IR images cannot display the device’s water content distribution properly. This is because the device has just been taken out of the oven, so the temperature of the device is high.

After the LiCl/PVA is printed onto the PFEEG, the device is placed in an oven and dried at 40 °C for 20 min to obtain the as-prepared PFEEG. As shown in **Figure S12a(i)**, the LiCl/PVA layer on the PFEEG exhibits excellent water retention performance after the as-prepared PFEEG is dried for 20 min (40 °C), attributed to the good hygroscopicity of LiCl/PVA. When the as-prepared PFEEGs are exposed to different humidity conditions, the initial ion concentration in the PFEEGs exhibits no significant variation, resulting in similar maximum output voltages. With the continuous voltage output, the ion concentration in the PFEEG decreases while the corrosion of the Fe-C negative electrode intensifies as RH increases, leading to variations in the duration of stable voltage output (**Figure 2a-b**). Furthermore, when the drying time of PFEEG at 40 °C is increased by 3 times (60 min), the water content of LiCl/PVA can continue to decrease (**Figure S12a(ii)**). As a result, when the as-prepared PFEEGs are placed under different humidity conditions (20%, 50%, and 90% RH), there is a significant difference in ion concentration in the device, resulting in an increase in the output voltage of the device as the humidity decreases (**Figure S12b-c**). As shown in **Figure S12d**, the IR images exhibit the change of the water content gradient inside the device when the device is dried at 40 °C for 20 min and then exposed at 90% RH for 0, 1, 6, 12, and 24 h, respectively. These IR images indicate the obvious water content gradient inside the device, with capillary water flow gradually transporting from the hygroscopic part to the non-hygroscopic part of the device as the exposure time under 90% RH increases. In addition, as shown in **Figure S29**, during electricity generation, ions obviously migrate from the hygroscopic part to the non-hygroscopic part, further demonstrating the existence of the water content gradient inside the device.


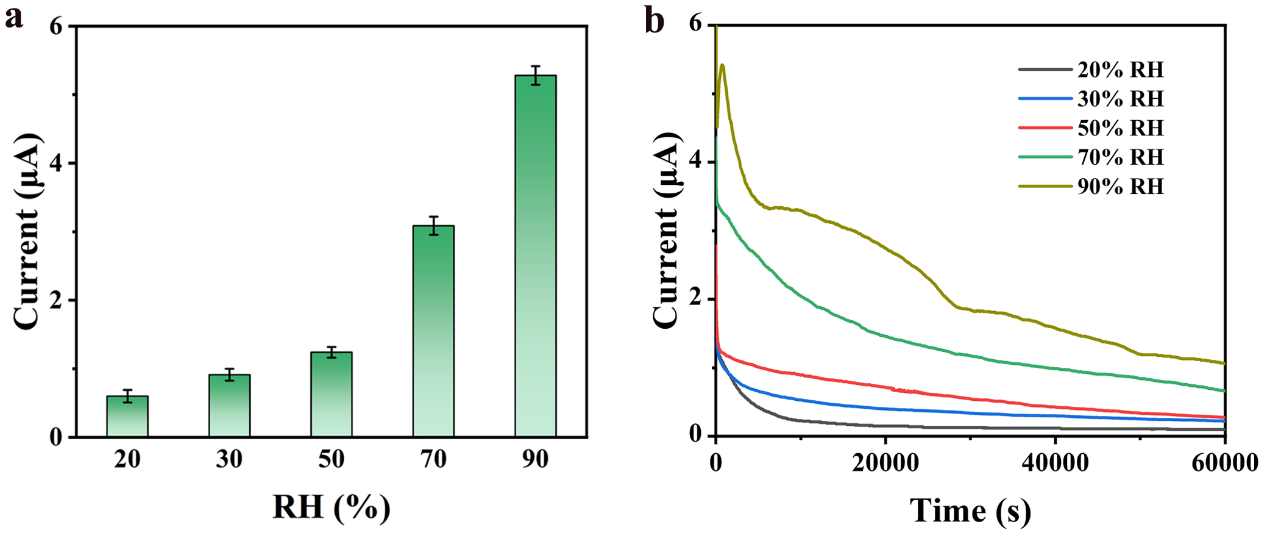


**Figure S13.** (a) Current and (b) continuous current output of the PFEEG under various humidity conditions (20~90% RH).


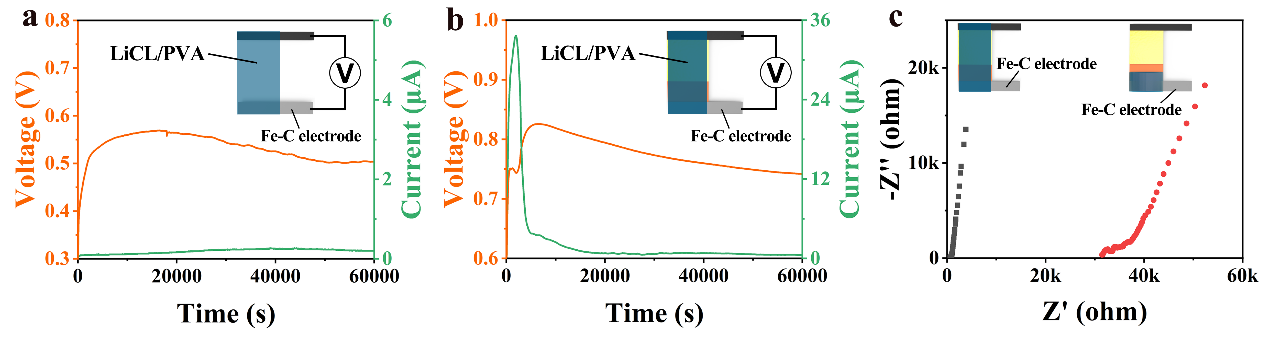


**Figure S14.** Oxidation-reduction reaction of Fe-C electrode to generate electricity. (a) The asymmetric electrode is infiltrated with LiCl/PVA. (b) The entire PFEEG is infiltrated with LiCl/PVA. (c) Electrochemical impedance spectra of fully LiCl/PVA-infiltrated PFEEG and Device 2. The frequency range was 0.01~105 Hz.

As shown in **Figure S14a**, when only the negative electrode (Fe-C electrode) and the positive electrode (C electrode) are infiltrated with LiCl/PVA, the maximum output voltage is about 0.57 V, then decreasing to about 0.51 V after continuous power generation for 60000 s. Moreover, the maximum output current only reach ~0.27 μA. As shown in **Figure S14b**, when the entire PFEEG is infiltrated with LiCl/PVA, the output voltage initially increases rapidly to about 0.83 V, and then gradually decreases to about 0.74 V. The device exhibits an instantaneous output current of about 33.65 μA, which rapidly decays to less than 1 μA within 16000 s. The reason for the higher maximum output current of the fully LiCl/PVA-infiltrated device compared to the PFEEG in normal operation is that the ionic conductivity of the fully infiltrated device is significantly greater than that of the PFEEG in normal operation (**Figure S14c**).^[8]^


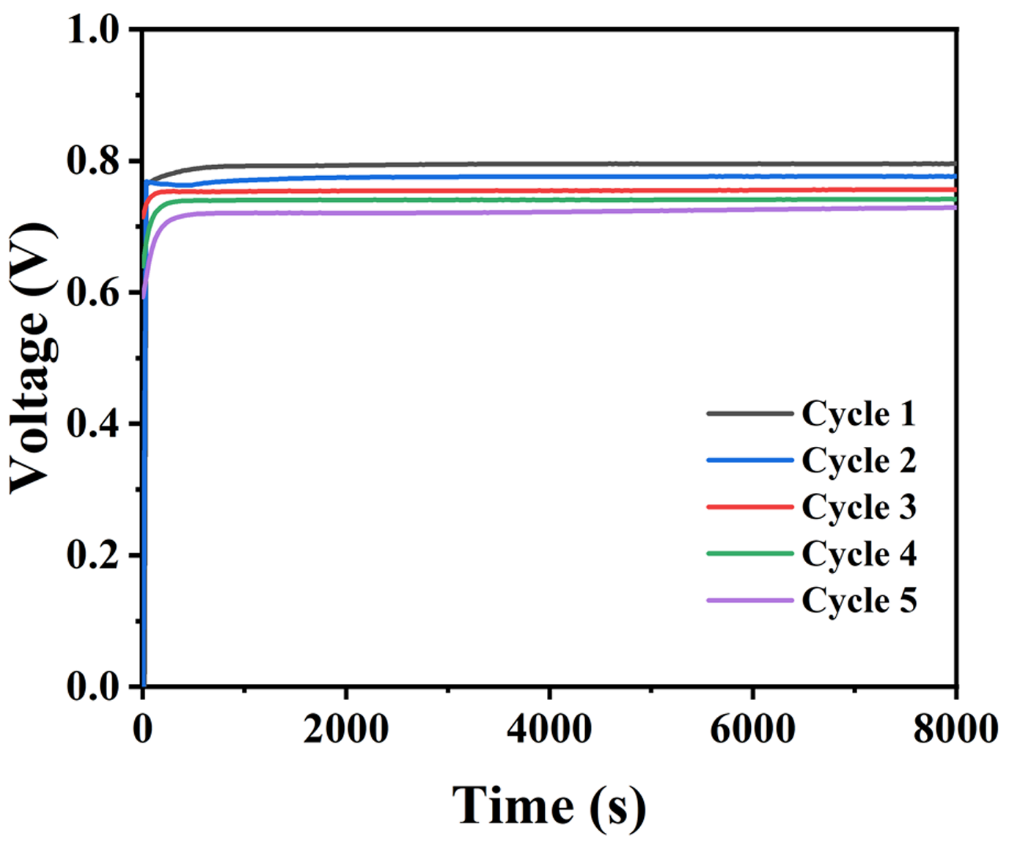


**Figure S15.** Repeatable electricity generation of the PFEEG. The PFEEG is exposed to air until stable, generating electricity for 8000 s, and then dried at 60 °C for 20 min. The tests are carried out at 50% RH.


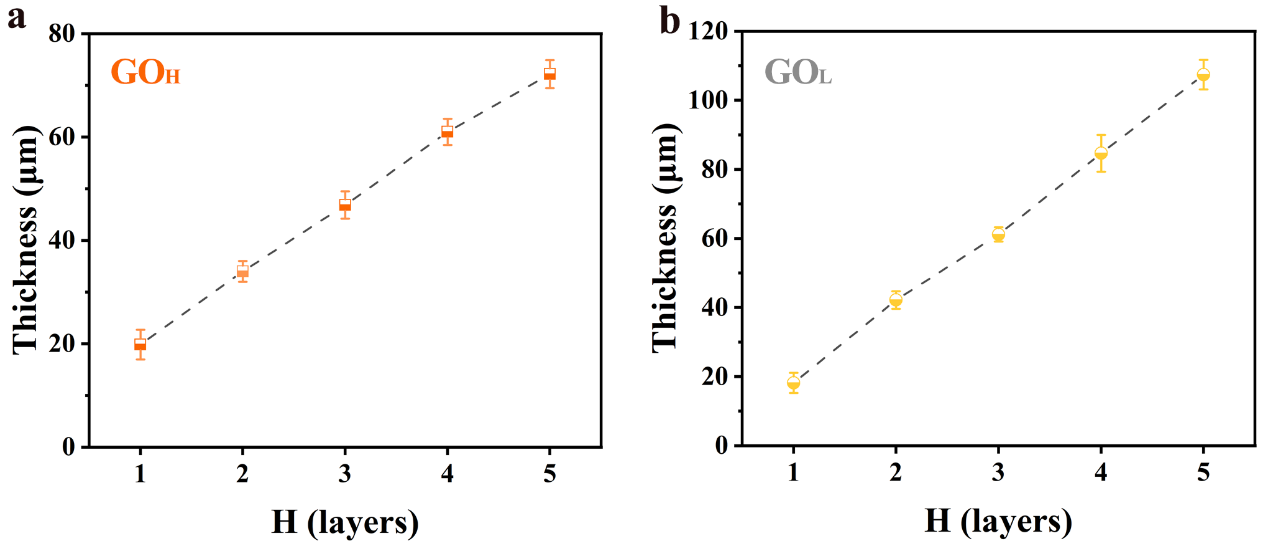
**Figure S16.** The screen-printed thicknesses of the (a) GO_H_ and (b) GO_L_ as a function of the number of the printing layers (H).


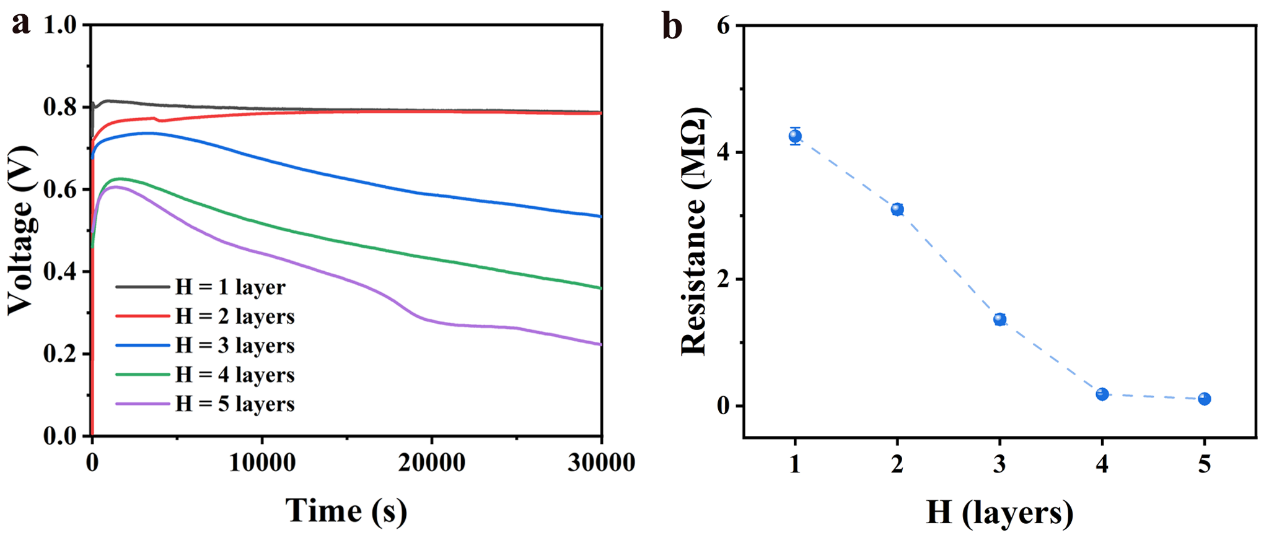


**Figure S17.** (a) Voltage output and (b) resistance variation of the PFEEG with different H (D_1_ = 4 mm and D_2_ = 2 mm).


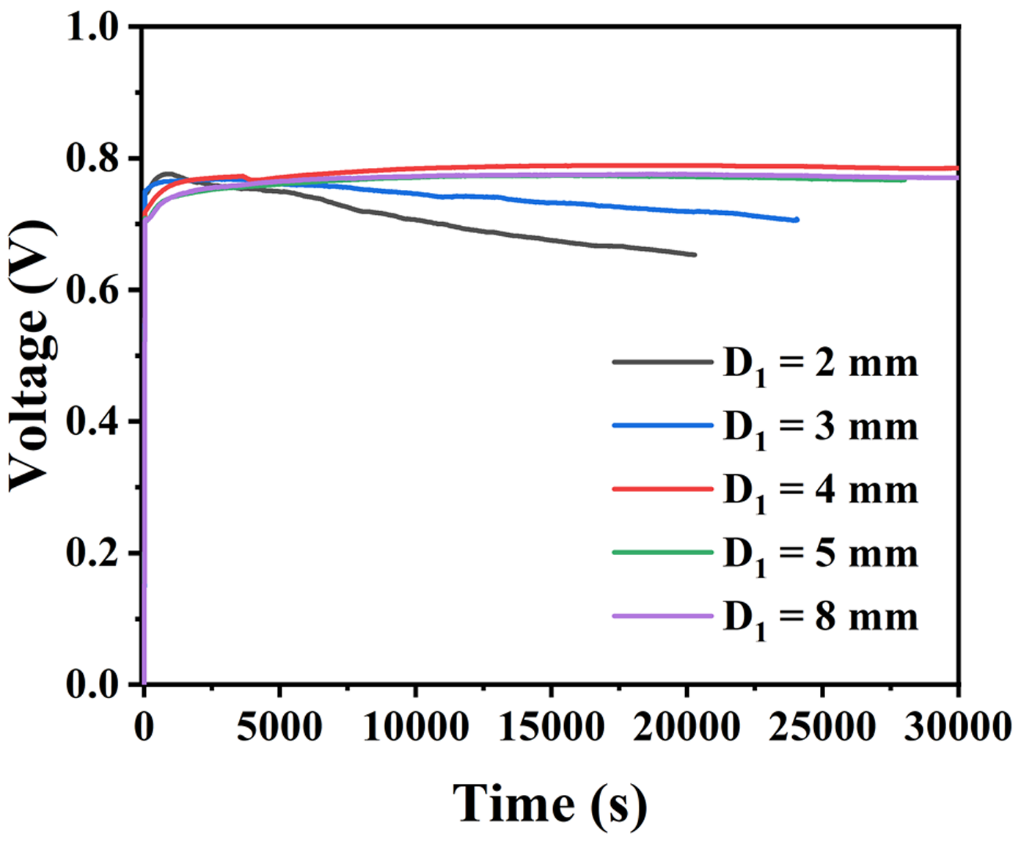


**Figure S18.** Voltage output of the PFEEG with different D_1_ (D_2_ = 2 mm and H = 2 layers).


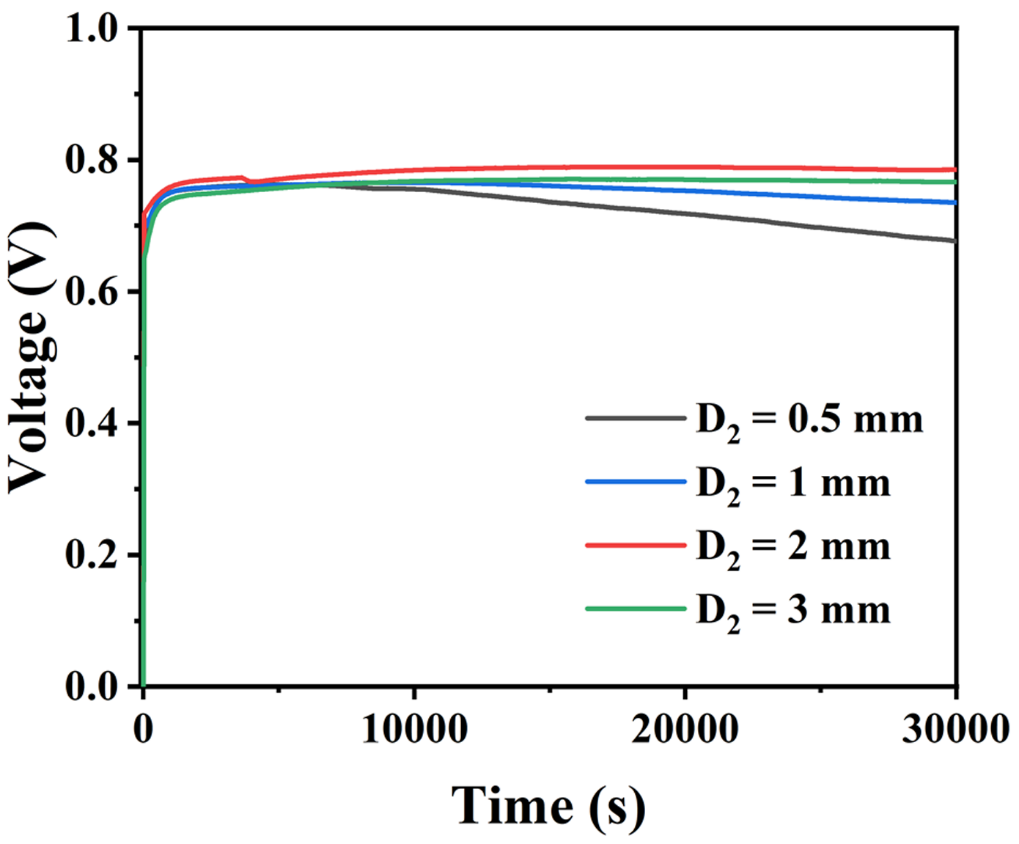


**Figure S19.** Voltage output of the PFEEG with different D_2_ (D_1_ = 4 mm and H = 2 layers).


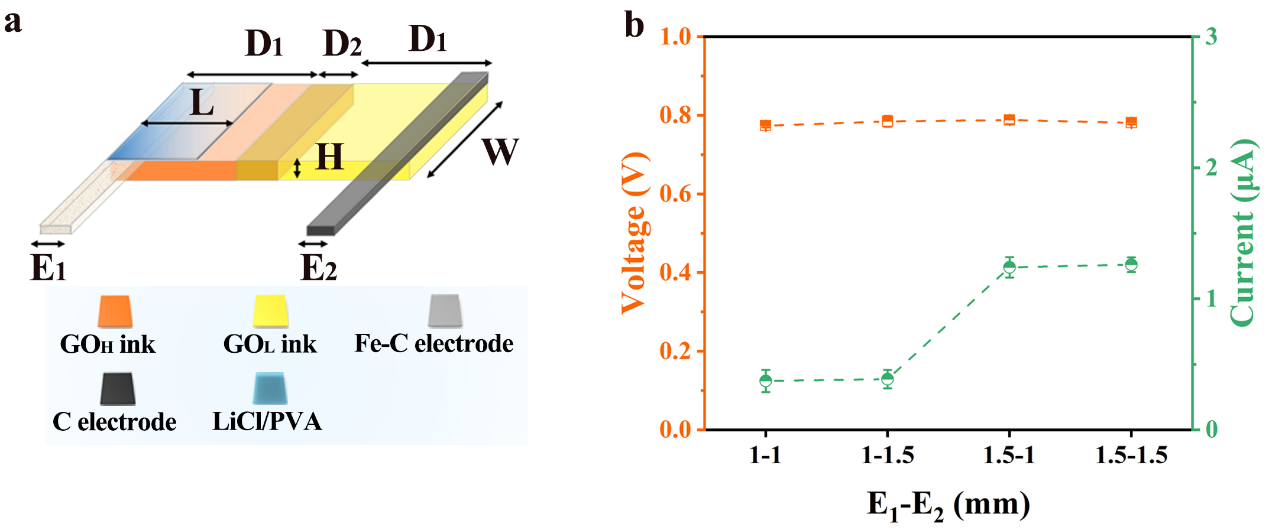


**Figure S20.** (a) Schematic of the PFEEGs with different printed parts. (b) Electric output of the PFEEG at different overlap widths of the negative and positive electrodes with GO_H_ and GO_L_ (E_1_ and E_2_).


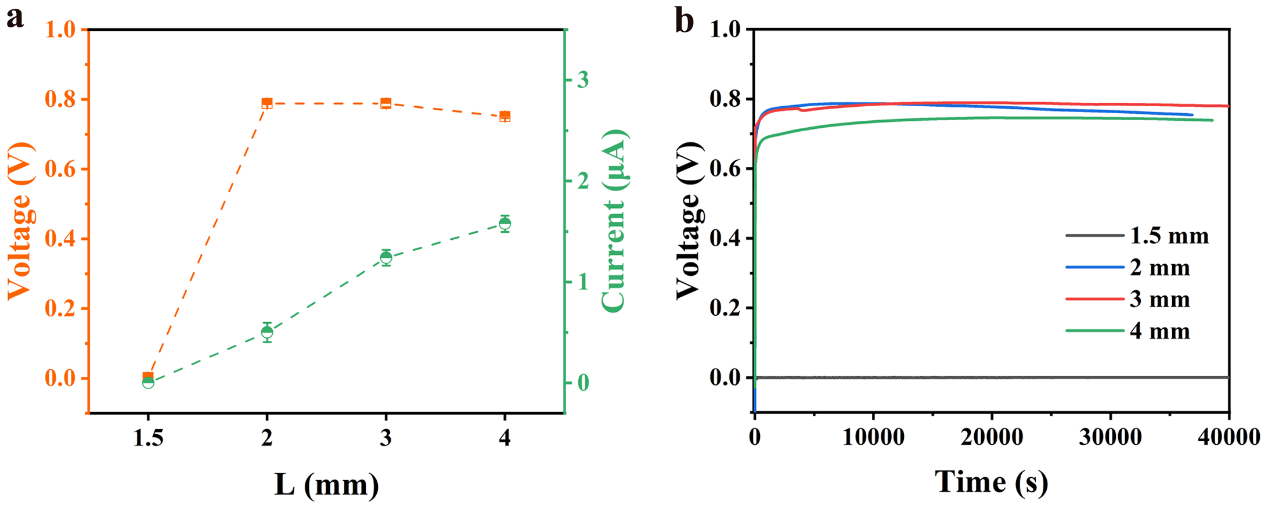


**Figure S21.** (a) Electric output and (b) voltage output of the PFEEG at different LiCl/PVA widths (L).


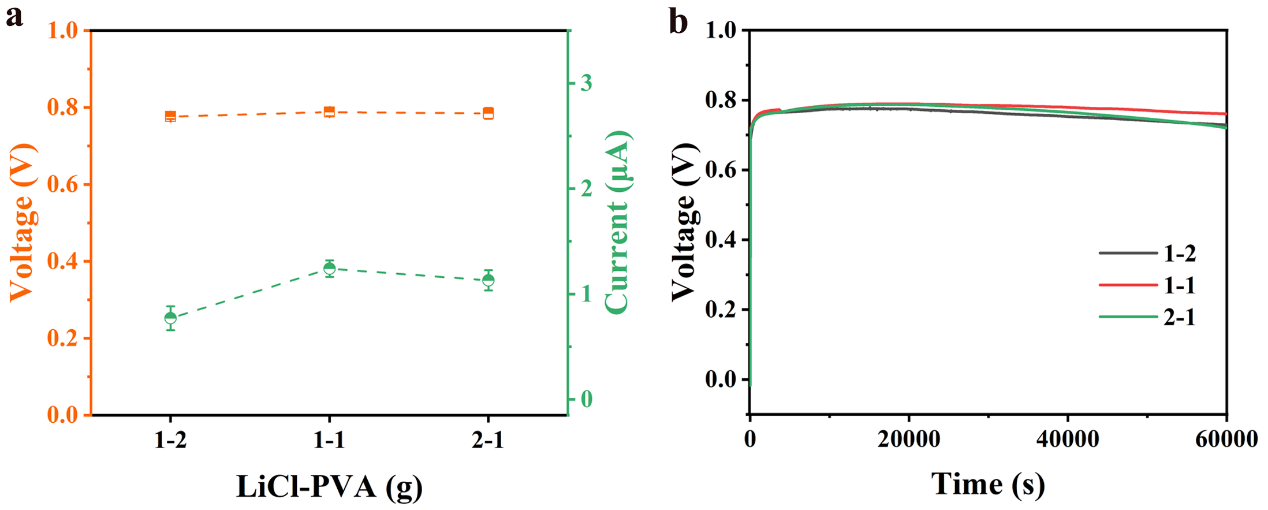


**Figure S22.** (a) Electric output and (b) voltage output of the PFEEG at different LiCl content.


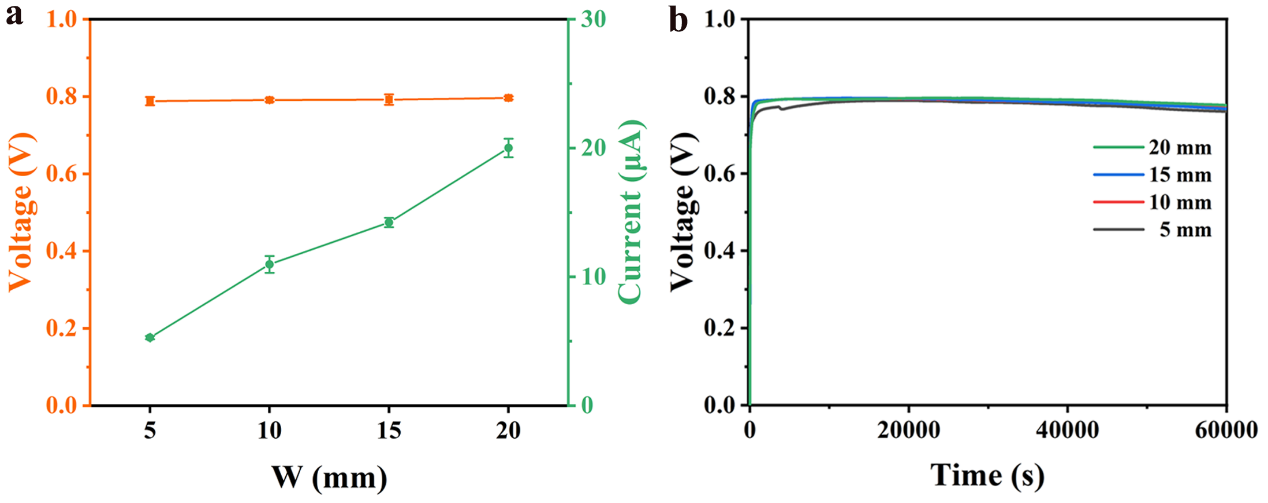


**Figure S23.** (a) Electric output and (b) voltage output of the PFEEG at different widths (W). The current output test is conducted at 90% RH.


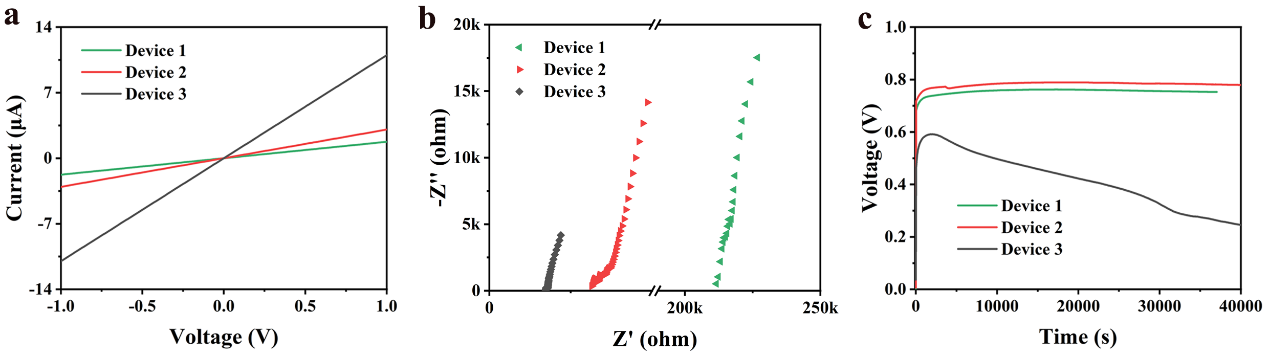


**Figure S24.** (a) I-V curves of Device 1, Device 2, and Device 3 (without LiCl/PVA) with the resistance of 568.61, 325.91, and 90.83 kΩ. (b) Electrochemical impedance spectra of Device 1, Device 2, and Device 3. The electrochemical impedance spectra are tested by a CHI760E electrochemical workstation. The frequency range was 0.01~10^5^ Hz. (c) Voltage output of these three devices.

The I-V curves of these three devices are measured by applying a voltage source with the scan rate of 10 mV s^-1^ at a voltage ranging from -1 V to 1 V. Based on the I-V curves, these three devices exhibit Ohmic contact behaviour. The electrical data was acquired using the Keithley 2461 sourcemeter controlled by the software developed by Wuhan Zeal Young Technology Co., Ltd.


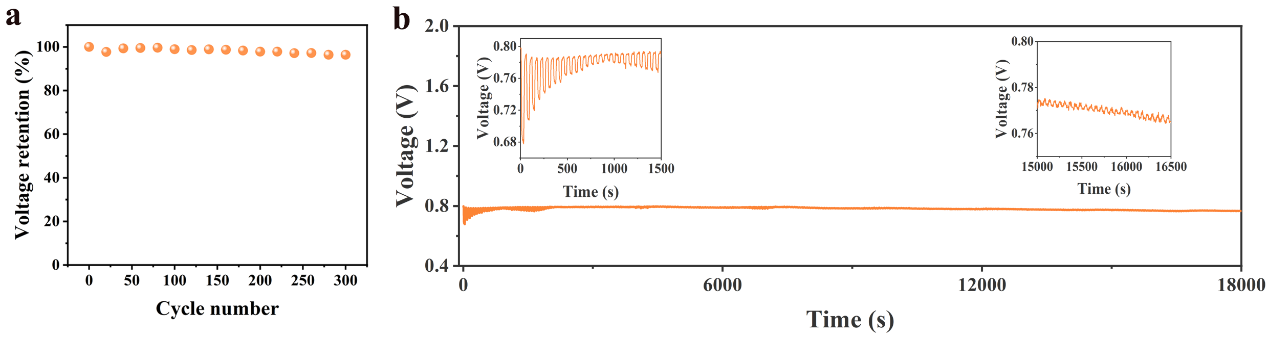


**Figure** **S25.** (a)Voltage retention and (b) voltage output of the PFEEG after various bending cycles at an angle of 150°.


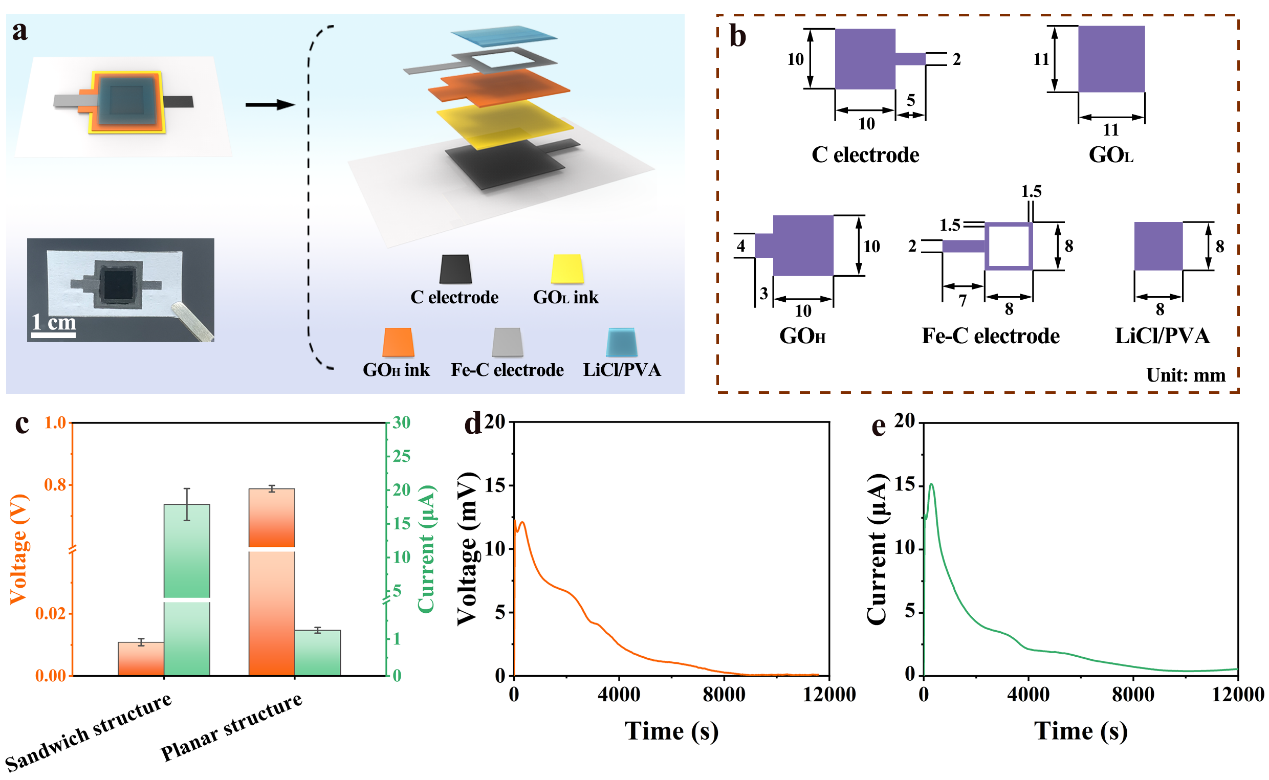


**Figure S26.** (a) Schematic illustration of the standard sandwich structure and the actual photo (inset) of a sandwich-structured PFEEG unit. (b) Size parameters of 5 parts of a standard sandwich-structured PFEEG. (c) Electric output of the sandwich-structured PFEEG and the planar-structured PFEEG. (d) Voltage output and (e) current output of the sandwich-structured PFEEG.


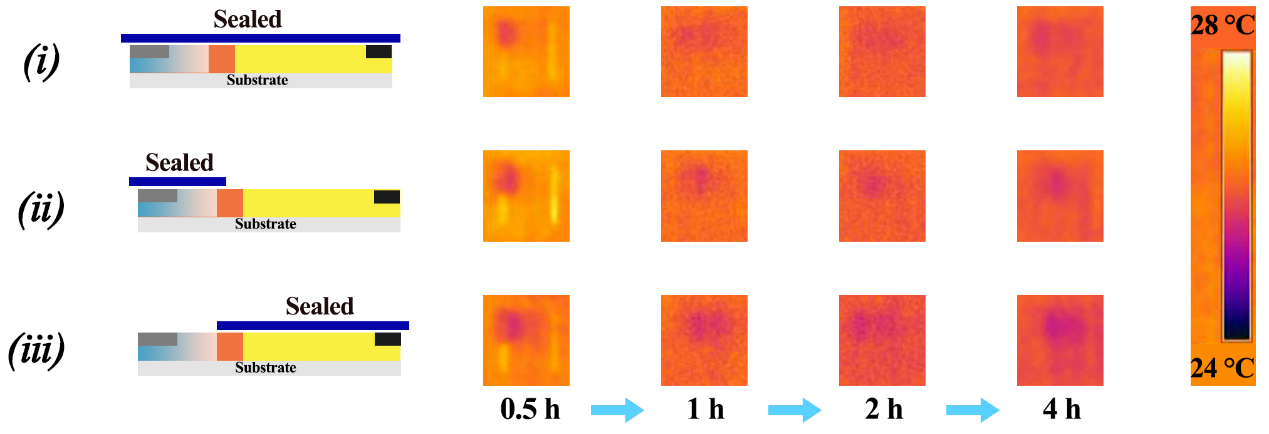


**Figure S27.** IR images of the internal capillary water flow at 0.5, 1, 2, and 4 h when (*ⅰ*) the PFEEG is completely sealed, (*ⅱ*) only the hygroscopic part is sealed, and (*ⅲ*) only the non-hygroscopic part is sealed, respectively.

When PFEEGs are placed in three sealed states ((*ⅰ*), (*ⅱ*), and (*ⅲ*)) for 0.5 h, the hygroscope part of (*ⅲ*) has more water content than that of (*ⅰ*) and (*ⅱ*). Because it can continuously absorb moisture from the ambient environment. After being placed for 4 h, the PFEEGs in (*ⅰ*) and (*ⅱ*) contain less water, while the PFEEG in (*ⅲ*) still contains more water.


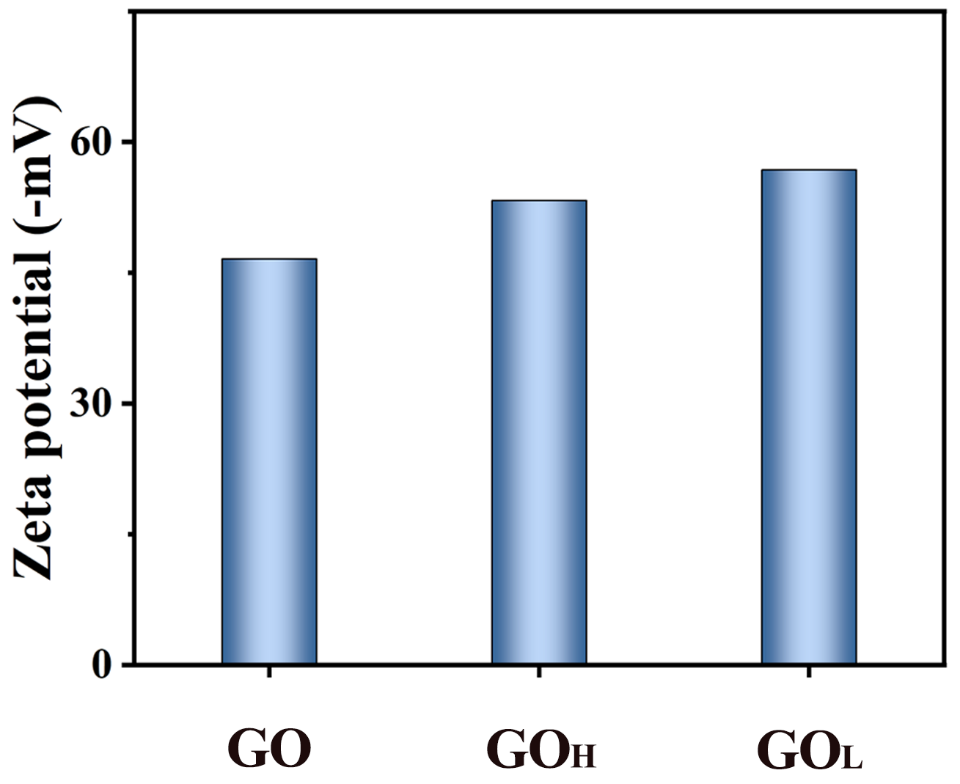


**Figure S28.** Zeta potential of GO, GO_H_, and GO_L_. Measurements are made on a Zetasizer Nano ZSP (Malvern Instruments, Malvern UK) using a detection angle of 173° at a temperature of 25 °C. The Nano ZSP uses a 10 mW He-Ne laser operating at a wavelength of 633 nm.


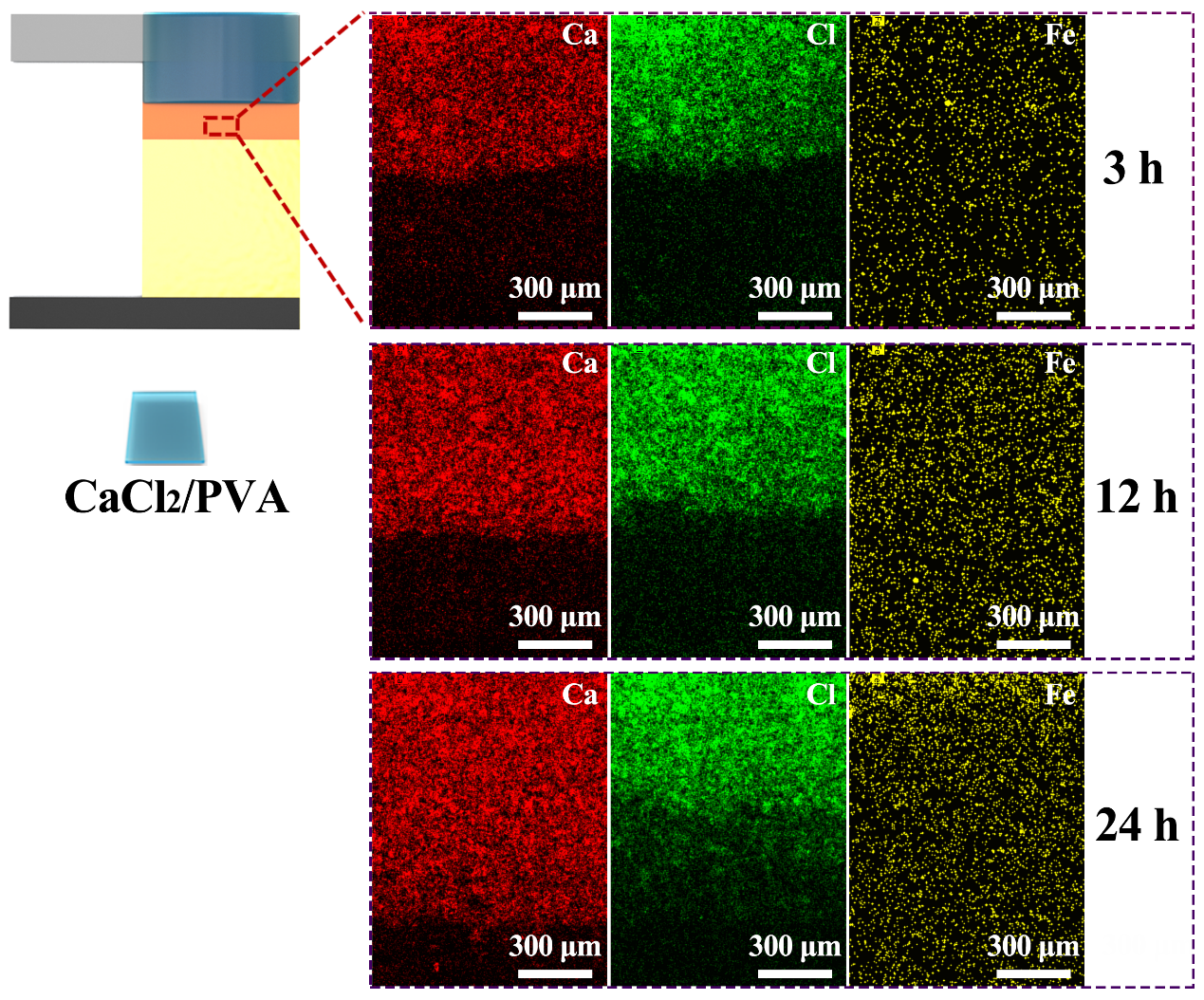


**Figure S29.** EDS element mapping images of calcium, chlorine, and iron in the non-hygroscopic part during the electricity generation (3 h, 12 h, and 24 h).

To clearly characterize the ion selectivity, CaCl_2_ is chosen to replace LiCl for investigating the ion selectivity of the PFEEG. By tracking the EDS of calcium, chlorine, and iron in the non-hygroscopic part, the distribution of CaCl_2_ during electricity generation of the PFEEG is revealed. After working for 3 h, only a small amount of calcium ions and chloride ions appears in the non-hygroscopic part, with no significant ion selectivity difference observed. After working for 12 h, the concentration of calcium and chloride ions in the non-hygroscopic part begins to increase. Due to the ion selectivity of the negatively charged channels, there is a notable repellant of chloride ions in the non-hygroscopic part, hindering their diffusion. After working for 24 h, the concentration of calcium ions in the non-hygroscopic part further increases, while the diffusion of chloride ions is markedly repelled. In addition, iron ions generated from the redox reaction of Fe in the negative electrode start to appear in the non-hygroscopic part. These results indicate that calcium ions and iron ions slowly migrate from the hygroscopic part to the non-hygroscopic part, demonstrating that the PFEEG has high ion selectivity.


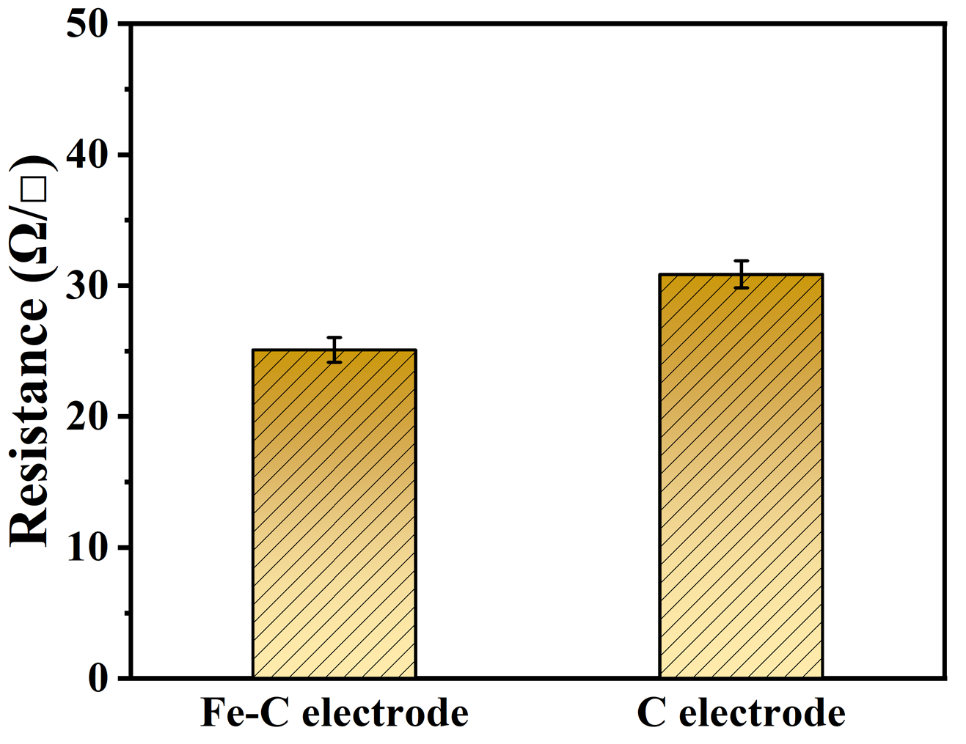


**Figure S30.** The resistances of the Fe-C electrode and the C electrode.


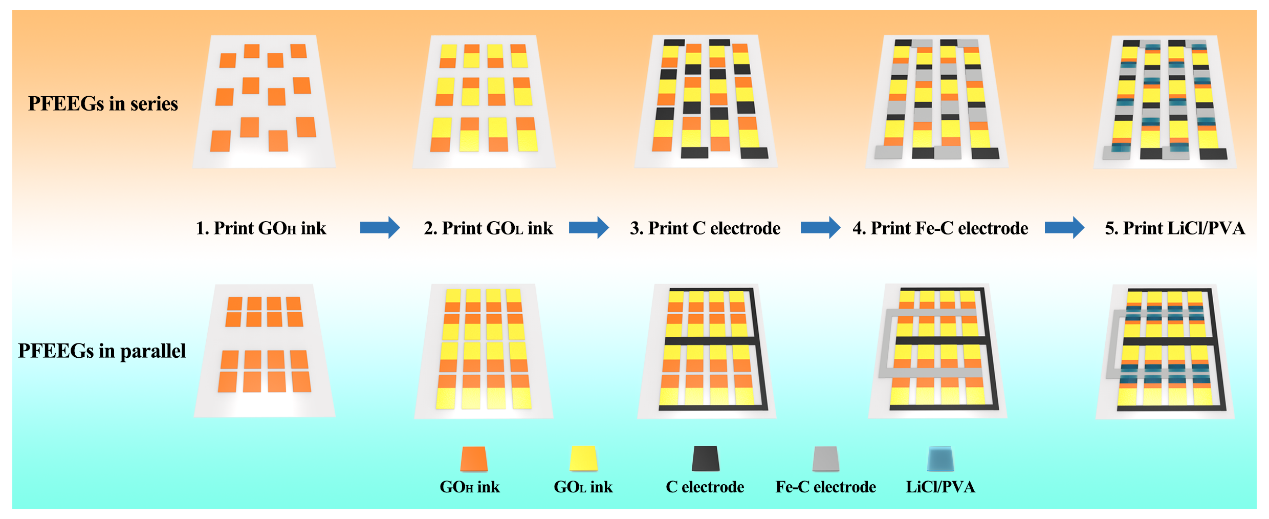


**Figure S31.** Schematic illustration of the large-scale fabrication of PFEEG arrays in serial and parallel connections by screen printing.


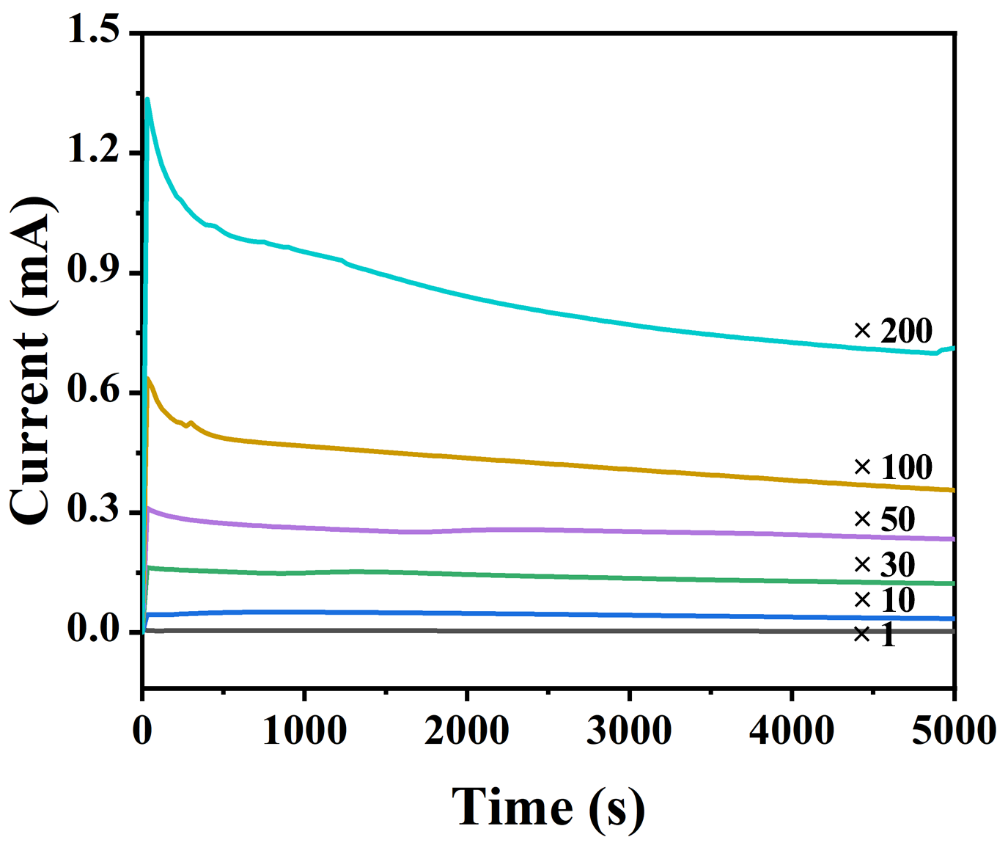


**Figure S32.** Current output of PFEEG arrays with different parallel numbers (90% RH).


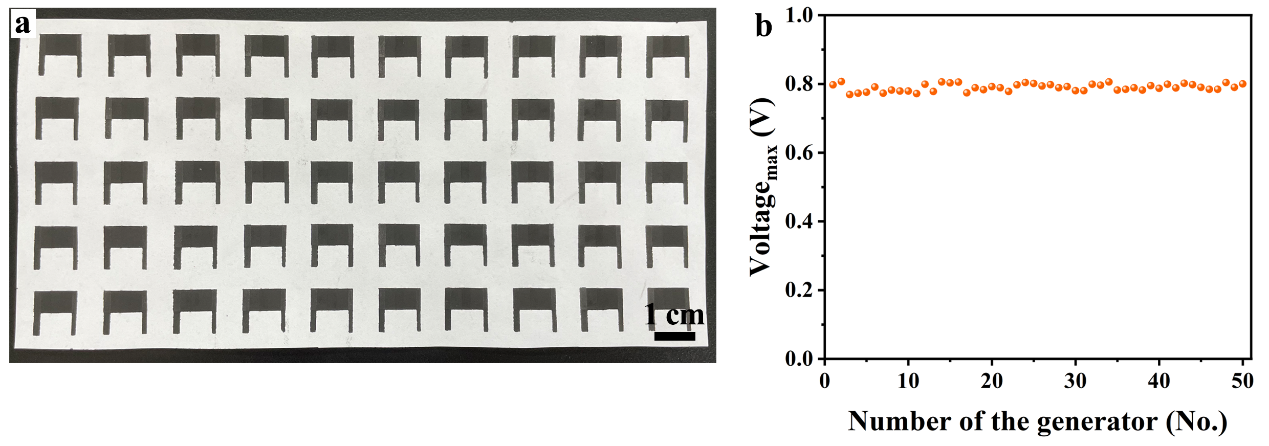


**Figure S33.** (a) The photo and (b) the maximum output voltage values of 50 PFEEGs printed on the copy paper substrate.


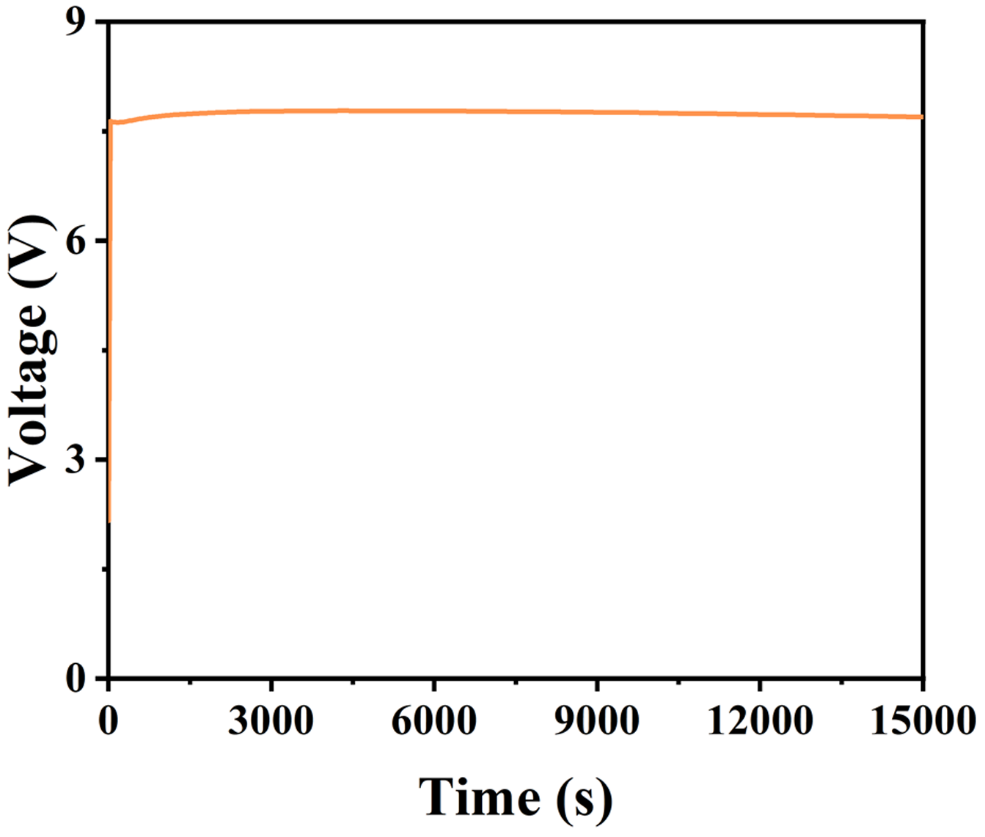


**Figure S34.** The voltage output of ribbon-shape PFEEGs with 10 serial connections during 500 bending cycles.


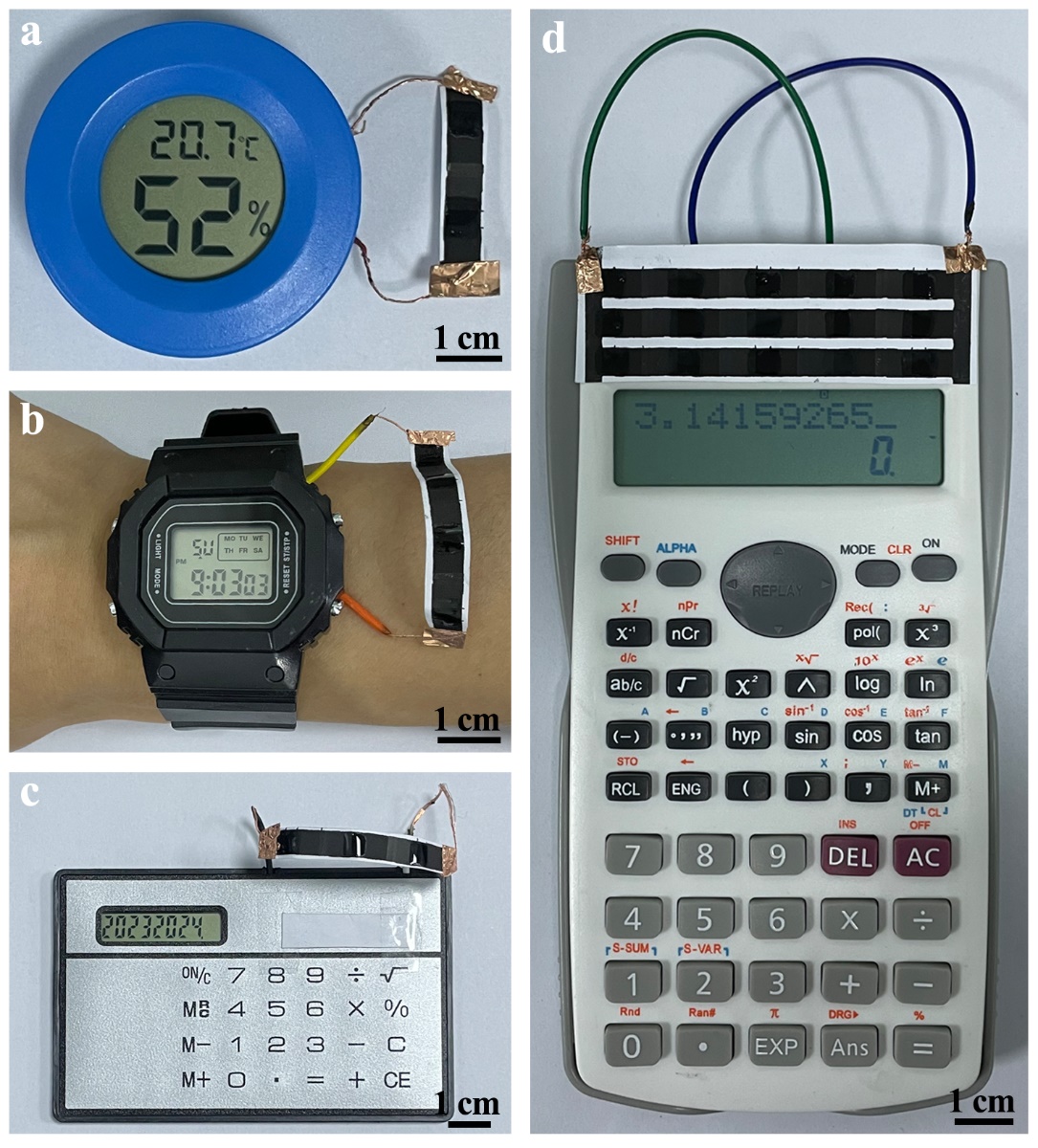


**Figure S35.** The drive of portable and wearable electronic devices by PFEEGs. (a) 2 PFEEGs in series can directly drive a commercial thermo-hygrometer. 3 PFEEGs in series can directly drive (b) an electronic watch or (c) a small commercial calculator. (d) 5 × 3 PFEEGs in series and parallel can directly drive a commercial scientific calculator.


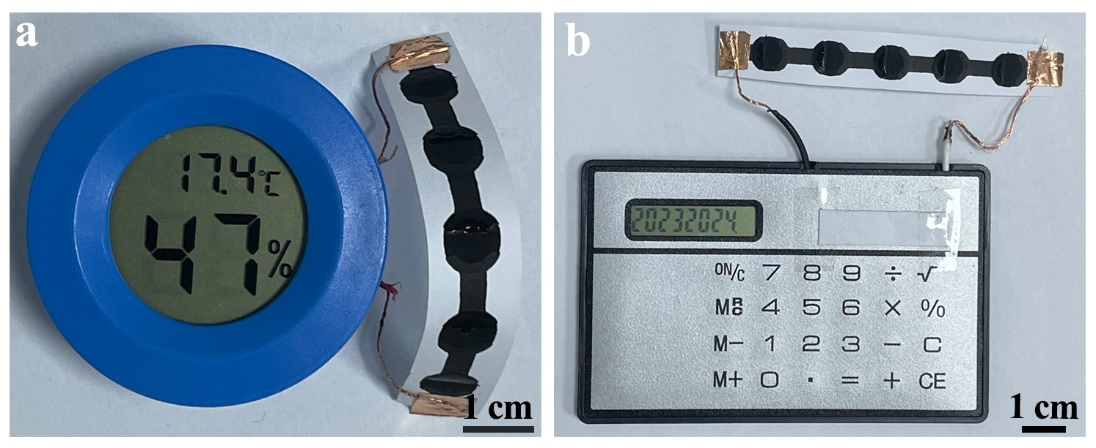


**Figure S36.** 5 circular PFEEGs in series can directly drive (b) a commercial thermo-hygrometer or (c) a small commercial calculator.


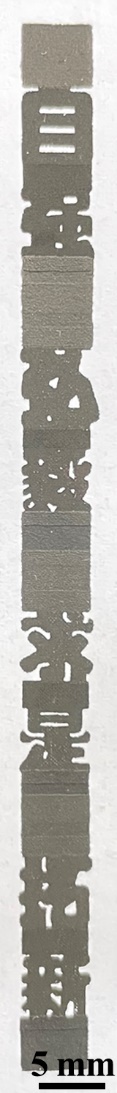


**Figure S37.** The photo of 4 serial PFEEGs composed of 8 Chinese characters of our school motto.


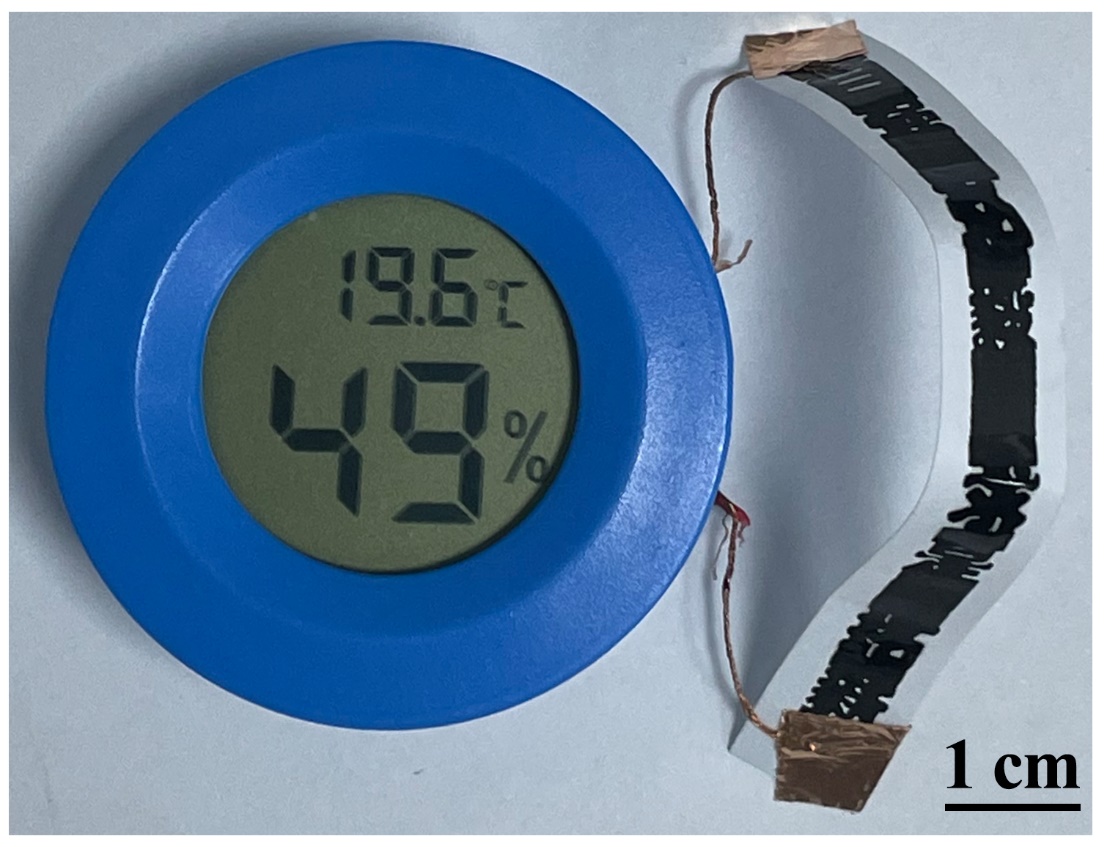


**Figure S38.** 4 series-connected PFEEGs of 8 Chinese characters of our school motto can directly drive a commercial thermo-hygrometer.


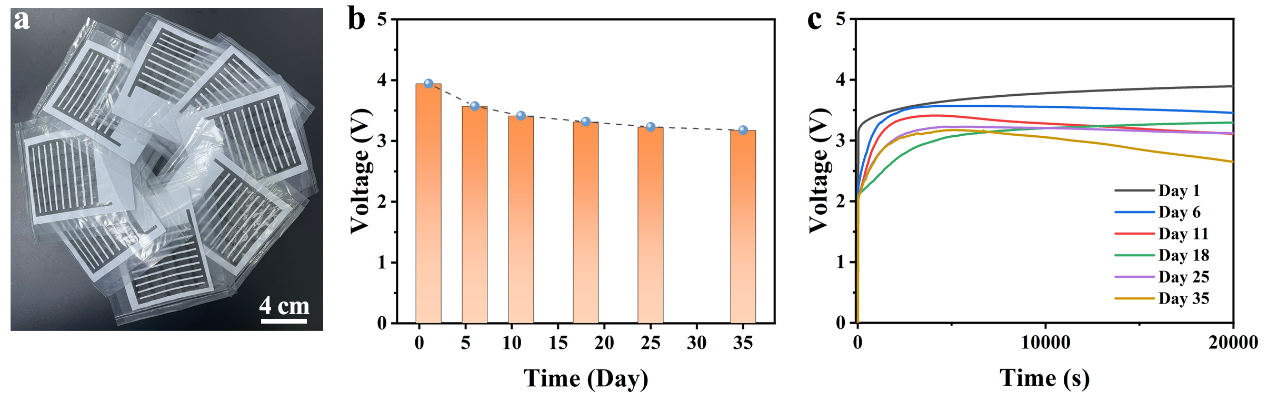


**Figure S39.** Voltage output of the power source unit (PSU) consists of 5 × 10 PFEEGs in series and parallel at different storage times. (a) Photo of the PSUs encapsulated in vacuum-packed bags. Voltage output (b) values and (c) curves of the PSU at different storage times (70% RH).


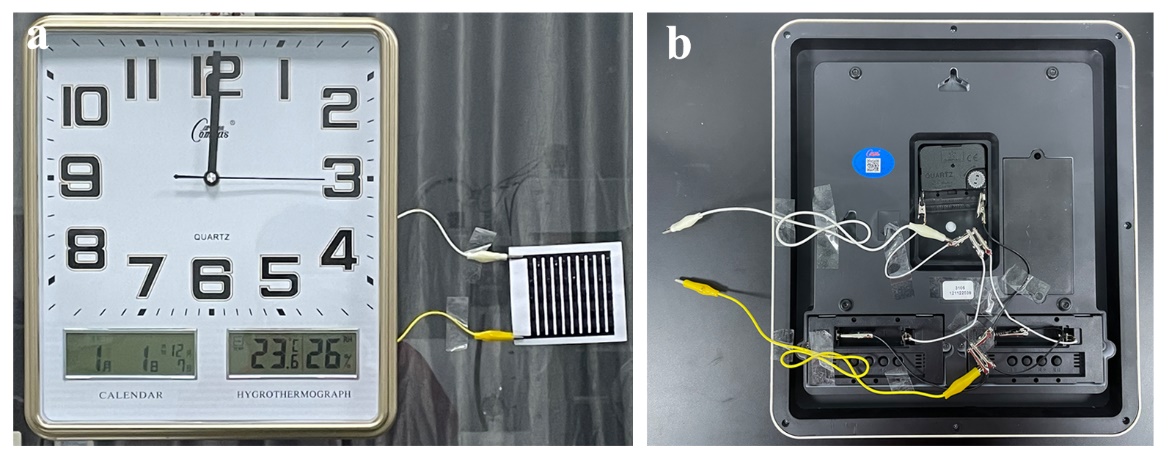


**Figure S40.** Photos of (a) a commercial clock (29 cm × 33 cm) in parallel with a calendar and a thermo-hygrometer driven directly by a PSU and (b) the circuit connection of the commercial clock in parallel with a calendar and a thermo-hygrometer.


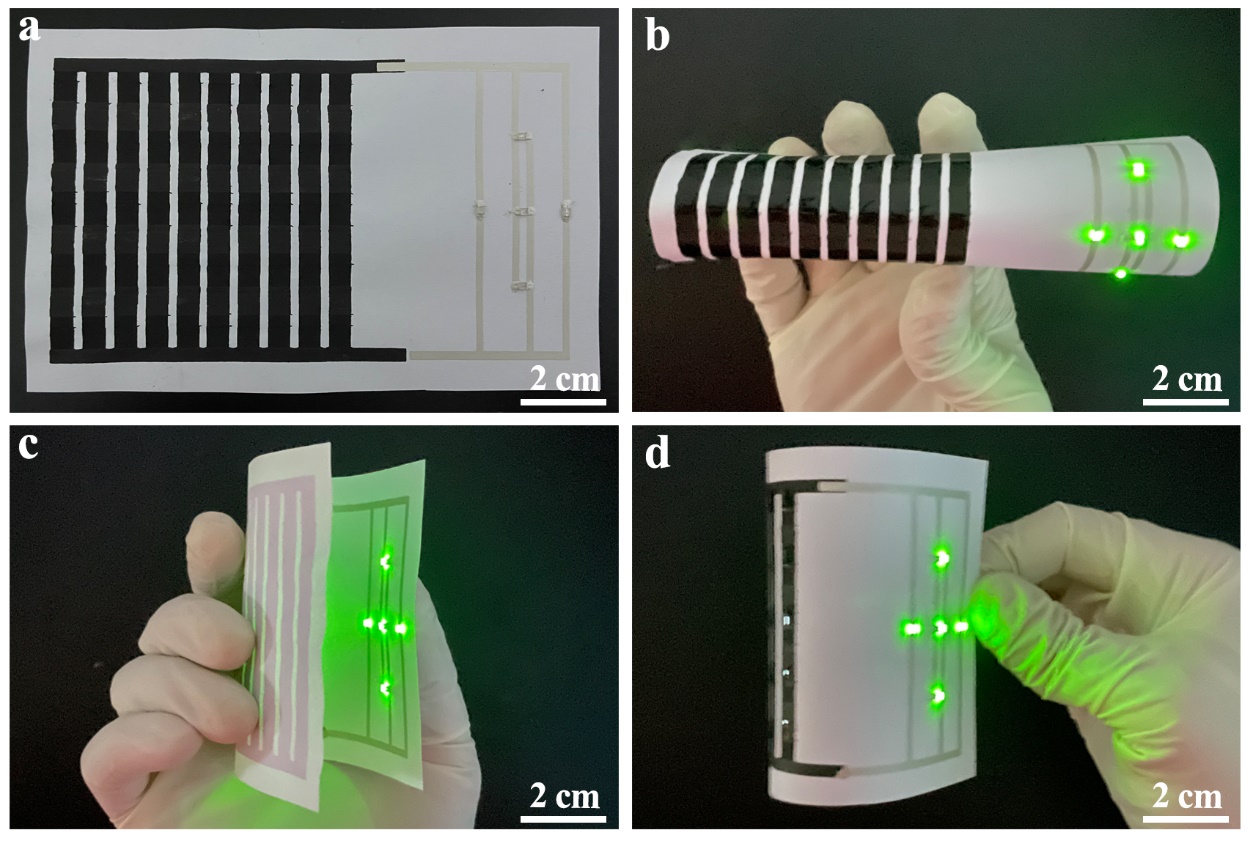


**Figure S41.** (a) Photo of the integrated system with the PFEEG array and a printed flexible circuit fabricated on a copy paper. (b-d) The circuit works successfully on different bent states.


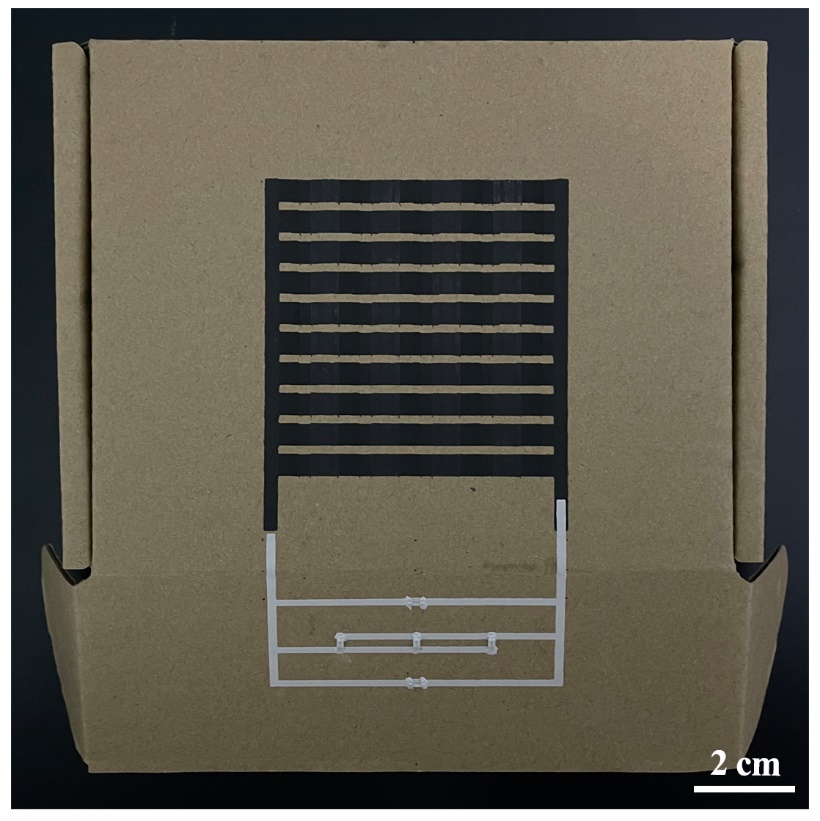


**Figure S42.** Photo of the flexible circuit printed on a corrugated cardboard box.


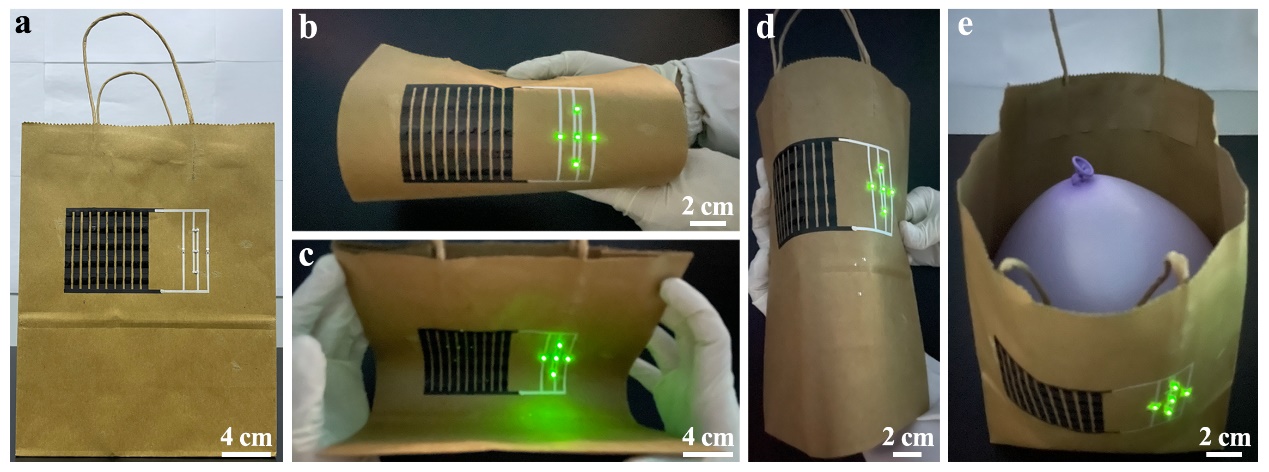


**Figure S43.** (a) Photo of the flexible circuit printed on a kraft paper shopping bag. (b-e) The circuit works successfully on different bent states.


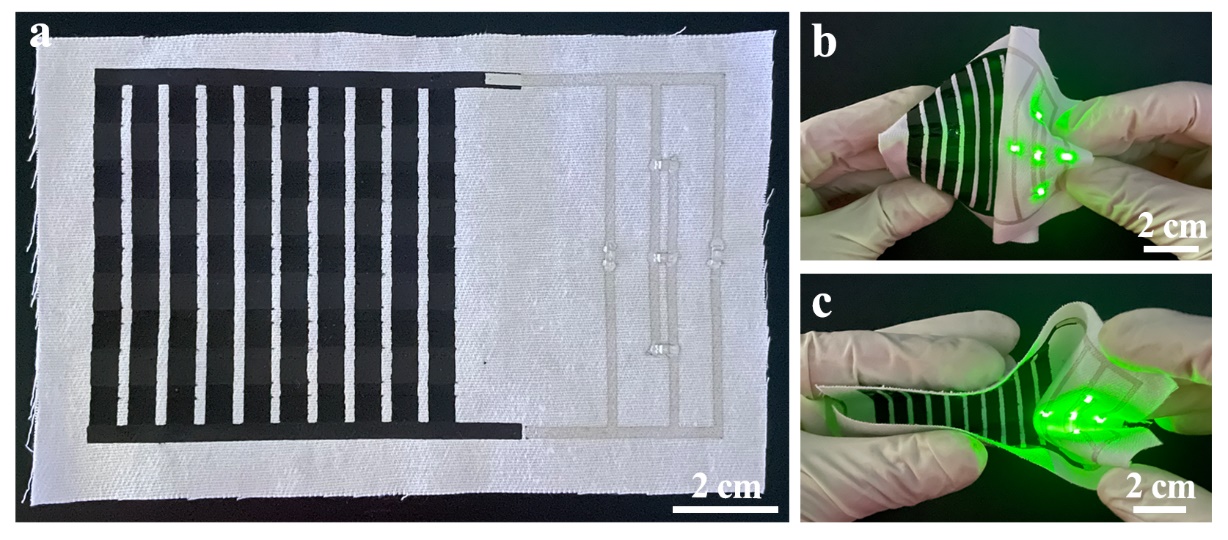


**Figure S44.** (a) Photo of the flexible circuit printed on a cotton fabric substrate. (b-c) The circuit works successfully on different bent states.


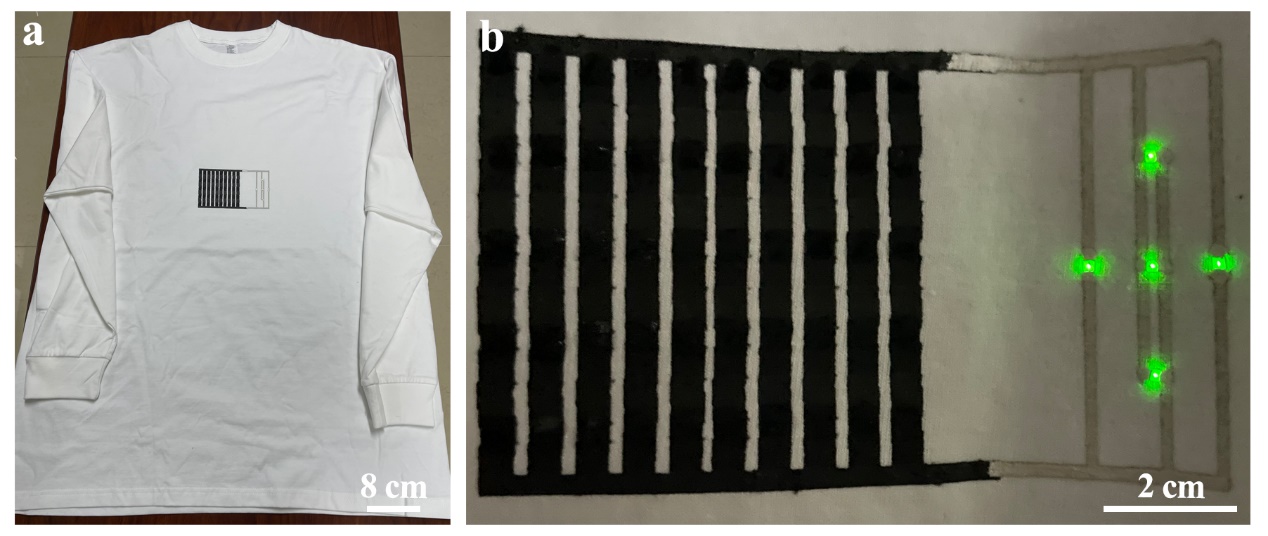


**Figure S45.** Photo of the flexible circuit (a) printed on a cotton T-shirt and (b) works successfully on a flat state.


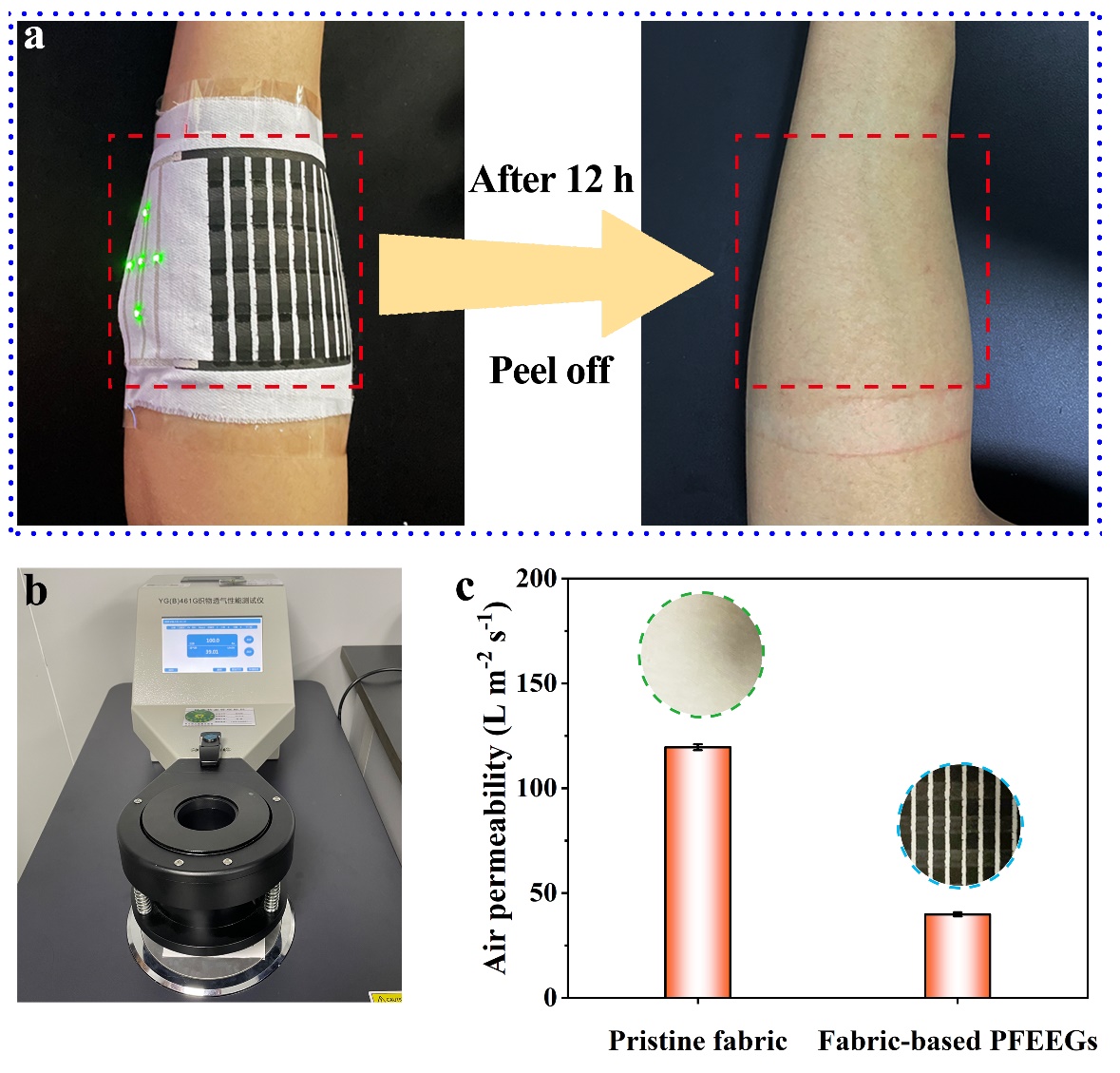


**Figure S46.** (a) Biocompatibility and safety test of the fabric-based PFEEGs. (b) Fabric air permeability is measured by a fabric permeability tester (YG(B)461G). The machine orifice area is 20 cm^2^, and the air permeability is tested at a pressure of 100 Pa. (c) Air permeability of the pristine fabric and the fabric-based PFEEGs.


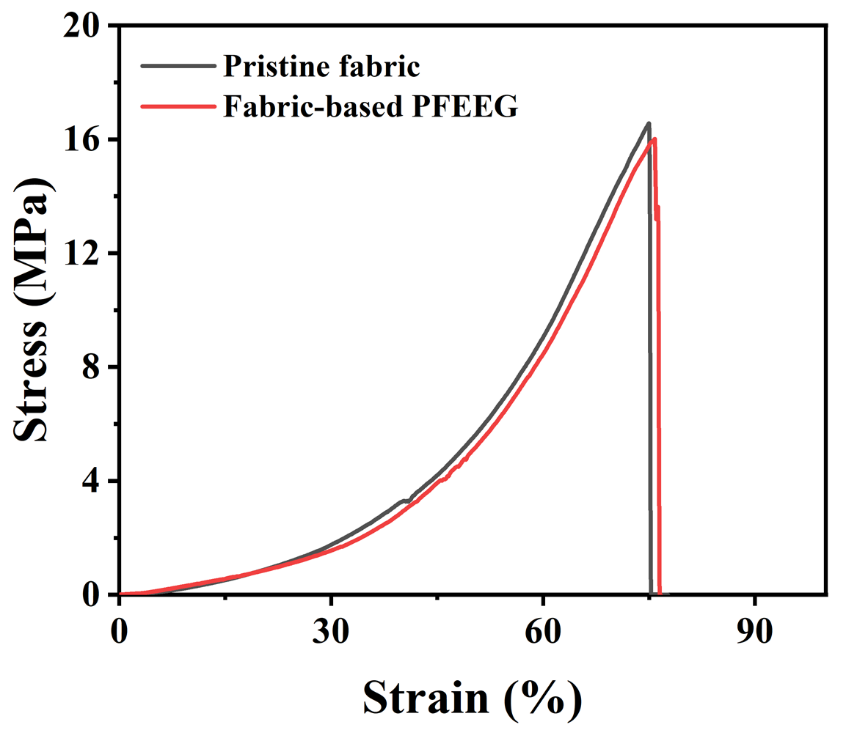


**Figure S47.** Stress-strain curves of the pristine fabric and the fabric-based PFEEG. The stretchability of the pristine fabric and the fabric-based PFEEG are measured by an electric tensile testing machine (ZQ-990LA-5).

**Table S1.** Summary and comparison of different flexible evaporation-driven generators.

| Material system | Water source | Voltage (V) | current density (mA cm^-3^) | Power density (μW cm^-2^) | Power density (mW cm^-3^) | Integration strategy | Reference |
| --- | --- | --- | --- | --- | --- | --- | --- |
| (GO/ANF-rGO/ANF)/CNT-PU | water reservoir | 0.96 | 0.068 |  | 0.07 | Manual assemble | ^[2]^ |
| RC/CNT fiber | Water droplet | 0.16 |  |  | 0.4 | Manual assemble | ^[9]^ |
| PEDOT:PSS/fabric | Water reservoir | 0.58 | 0.004 | 0.45 | 0.04 | Manual assemble | ^[10]^ |
| Si NWs | Water droplet | 1.5 | 0.06 | 1.60 | 0.16 | Manual assemble | ^[11]^ |
| PPy/TiO_2_ fabric | Water droplet | 0.65 | 0.04 |  |  | Manual assemble | ^[12]^ |
| CB/cotton fabric | Hygroscopic salt | 0.74 | 0.07 | 0.60 | 0.05 | Manual assemble | ^[13]^ |
| Ni-Al layered double hydroxide | Water reservoir | 0.6 | 0.0009 | 0.008 | 0.015 | Manual assemble | ^[14]^ |
| CB/cotton fabric | Water droplet | 0.53 | 0.01 | 0.01 | 0.0008 | Manual assemble | ^[15]^ |
| Ni-Al layered double hydroxide | Water reservoir | 0.7 | 0.12 | 0.01 | 0.016 | Manual assemble | ^[16]^ |
| GO/AC | Hygroscopic hydrogel | 0.8 | 2.76 | 1.55 | 0.41 | Screen printing | This work |

**Movie S1.** A clock (29 cm × 33 cm) in parallel with an electronic calendar and a thermo-hygrometer directly powered by a PSU.

**Movie S2.** Mechanical flexibility of the printed flexible circuit fabricated on a copy paper substrate.

**Movie S3.** Mechanical flexibility of the printed flexible circuit fabricated on a cotton fabric substrate.

**Movie S4.** Visual monitoring of human breathing rate by the printed self-powered visual sensing system.

**References**

[1] X. Zhang, M. Wang, Y. Wu, X. Chen, K. Wu, Q. Fu, H. Deng, *Adv. Funct. Mater.* ***2022***, *33*, 2210027.

[2] X. Zhang, X. Chen, Y. Qu, Y. Wu, K. Wu, H. Deng, Q. Fu, *Nano Energy* ***2022***, *98*, 107241.

[3] D. Lei, Q. Zhang, N. Liu, T. Su, L. Wang, Z. Ren, Z. Zhang, J. Su, Y. Gao, *Adv. Funct. Mater.* ***2021***, *32*, 2107330.

[4] T. He, H. Wang, B. Lu, T. Guang, C. Yang, Y. Huang, H. Cheng, L. Qu, *Joule* ***2023***, *7*, 935.

[5] H. Wang, Y. Sun, T. He, Y. Huang, H. Cheng, C. Li, D. Xie, P. Yang, Y. Zhang, L. Qu, *Nat. Nanotechnol.* ***2021***, *16*, 811.

[6] H. Cheng, Y. Huang, F. Zhao, C. Yang, P. Zhang, L. Jiang, G. Shi, L. Qu, *Energy Environ. Sci.* ***2018***, *11*, 2839.

[7] H. Cheng, F. Zhao, J. Xue, G. Shi, L. Jiang, L. Qu, *ACS Nano* ***2016***, *10*, 9529.

[8] J. Tan, S. Fang, Z. Zhang, J. Yin, L. Li, X. Wang, W. Guo, *Nat. Commun.* ***2022***, *13*, 3643.

[9] J. Chen, Y. Li, Y. Zhang, D. Ye, C. Lei, K. Wu, Q. Fu, *Adv. Funct. Mater.* ***2022***, *32*, 2203666.

[10] T. G. Yun, J. Bae, H. G. Nam, D. Kim, K. R. Yoon, S. M. Han, I.-D. Kim, *Nano Energy* ***2022***, *94*, 106946.

[11] B. Shao, Y. Wu, Z. Song, H. Yang, X. Chen, Y. Zou, J. Zang, F. Yang, T. Song, Y. Wang, M. Shao, B. Sun, *Nano Energy* ***2022***, *94*, 106917.

[12] J. Xie, Y. Wang, S. Chen, *Chem. Eng. J.* ***2021***, *431*, 133236.

[13] J. Bae, T. G. Yun, B. L. Suh, J. Kim, I.-D. Kim, *Energy Environ. Sci.* ***2020***, *13*, 527.

[14] J. Tian, Y. Zang, J. Sun, J. Qu, F. Gao, G. Liang, *Nano Energy* ***2020***, *70*, 104502.

[15] T. G. Yun, J. Bae, A. Rothschild, I. D. Kim, *ACS Nano* ***2019***, *13*, 12703.

[16] J. Sun, P. Li, J. Qu, X. Lu, Y. Xie, F. Gao, Y. Li, M. Gang, Q. Feng, H. Liang, X. Xia, C. Li, S. Xu, J. Bian, *Nano Energy* ***2019***, *57*, 269.
